# Higher sea surface temperature in the Indian Ocean during the Last Interglacial weakened the South Asian monsoon

**DOI:** 10.1073/pnas.2107720119

**Published:** 2022-03-01

**Authors:** Yiming V. Wang, Thomas Larsen, Stefan Lauterbach, Nils Andersen, Thomas Blanz, Uta Krebs-Kanzow, Paul Gierz, Ralph R. Schneider

**Affiliations:** ^a^Department of Archaeology, Max Planck Institute for the Science of Human History, 07745 Jena, Germany;; ^b^Leibniz Laboratory for Radiometric Dating and Stable Isotope Research, Kiel University, 24118 Kiel, Germany;; ^c^Institute of Geosciences, Kiel University, 24118 Kiel, Germany;; ^d^Helmoltz Center for Polar and Marine Research, Alfred Wegener Institute, 27578 Bremerhaven, Germany

**Keywords:** paleohydroclimate and paleoenvironment, Indian summer monsoon, Bay of Bengal, sedimentary leaf wax, compound specific isotopes

## Abstract

Addressing and anticipating future South Asian monsoon changes under continuing global warming is of critical importance for the food security and socioeconomic well-being of one-quarter of the world’s population. However, climate model projections show discrepancies in future monsoon variability in South Asian monsoon domains, largely due to our still limited understanding of the monsoon response to warm climate change scenarios. Particularly, climate models are largely based on the assumption that higher solar insolation causes higher rainfall during similar warm climatic regimes, but this has not been verified by proxy data for different interglacial periods. Here, we compare Indian summer monsoon (ISM) variability during the Last Interglacial and Holocene using a sedimentary leaf wax δD and δ^13^C record from the northern Bay of Bengal, representing the Ganges–Brahmaputra–Meghna (G-B-M) river catchment. In combination with a seawater salinity record, our results show that ISM intensity broadly follows summer insolation on orbital scales, but ISM intensity during the Last Interglacial was lower than during the Holocene despite higher summer insolation and greenhouse gas concentrations. We argue that sustained warmer sea surface temperature in the equatorial and tropical Indian Ocean during the Last Interglacial increased convective rainfall above the ocean but dampened ISM intensity on land. Our study demonstrates that besides solar insolation, internal climatic feedbacks also play an important role for South Asian monsoon variability during warm climate states. This work can help to improve future climate model projections and highlights the importance of understanding controls of monsoonal rainfall under interglacial boundary conditions.

## Significance

Understanding the drivers of South Asian monsoon intensity is pivotal for improving climate forecasting under global warming scenarios. Solar insolation is assumed to be the dominant driver of monsoon variability in warm climate regimes, but this has not been verified by proxy data. We report a South Asian monsoon rainfall record spanning the last ∼130 kyr in the Ganges–Brahmaputra–Meghna river catchment. Our multiproxy data reveal that the South Asian monsoon was weaker during the Last Interglacial (130 to 115 ka)—despite higher insolation—than during the Holocene (11.6 ka to present), thus questioning the widely accepted model assumption. Our work implies that Indian Ocean warming may increase the occurrence of severe monsoon failures in South Asia.

The South Asian monsoon, also known as Indian summer monsoon (ISM), is one of the world’s most sensitive weather systems (1, 2). It is also one of the most critical weather systems for human livelihood. The water and food security of billions of people on the Indian subcontinent and adjacent areas is under pressure by increased weather anomalies and extreme monsoonal rainfall events (1). Despite the far-reaching consequences, prediction of ISM behavior under climate warming scenarios remains a key challenge for both global and regional climate models (3–5). Due to inconsistencies in model projections, debates center on whether the ISM will weaken or strengthen in a warming climate (2–4, 6). In this regard, a major challenge is our incomplete understanding of the extent to which ISM intensity responds to rapidly changing climatic factors—such as rising sea surface temperature (SST), elevated atmospheric greenhouse gas (GHG) concentrations, changing vegetation cover, and decreasing ice sheets and sea ice cover—and their interactive dynamics in a warming climate (1, 2, 5, 7, 8). Several proxy records from stalagmites and marine sediment archives on and around the Indian subcontinent have extended instrumental records and helped to identify monsoon response and variability to various climate forcings (9–12). However, these studies have mainly focused on distinct climatic transitions, namely, between glacial and interglacial periods. While studies showed that the monsoon was generally stronger during warm interglacials and interstadials compared to cold glacials and stadials, the varying degree of monsoonal rainfall intensity between different warm periods—when climatic boundary conditions were fairly similar—is often overlooked. Obtaining and comparing climatic information from different warm periods is therefore highly relevant for constraining uncertainties in model projections for a future warming climate (13).

At orbital time scales, changes in incoming solar insolation are regarded the most prominent control for the difference between overall glacial and interglacial monsoon rainfall intensity because past fluctuations of monsoon strength coincide remarkably well with changes in the Earth’s precessional cycle (5, 8, 9, 14–17). Higher summer insolation leads to enhanced atmospheric humidity, wind circulation, and land–sea thermal gradients, which ultimately increase precipitation (8, 18). Although it is generally assumed that higher insolation during warmer interglacials also results in higher ISM rainfall intensity, solar modulation of monsoon intensity and variation during different interglacials has remained largely unexplored. The last interglacial period, commonly correlated with marine isotope stage (MIS) 5e, can be considered a good analog for future climate scenarios because ice sheets at that time were much smaller, while temperatures and sea level were higher than at present (19–21). Compared to the Last Interglacial, the present interglacial period, the Holocene, also underwent comparable changes in orbital configurations, although the magnitude of boreal summer insolation change was weaker at precessional perihelion conditions (22, 23). Because of the higher boreal summer insolation and global SST (by 1 to 2 °C) and the lower ice volume during the Last Interglacial than during the Holocene (24, 25), fully coupled global ocean–atmosphere climate models predict higher monsoon rainfall intensity during the Last Interglacial (ca. 130 to 115 ka) compared to the Holocene (11.6 ka to present) (13, 17, 18). However, despite such model predictions, no direct evidence for higher ISM intensity during the Last Interglacial than during the Holocene has been presented so far.

Last Interglacial rainfall reconstructions for the Bay of Bengal branch of the ISM are typically based on stalagmite stable oxygen isotope (δ^18^O) records, which show varying magnitudes of δ^18^O changes at different sites on the Indian subcontinent (9, 11). However, great caution should be taken in equating stalagmite δ^18^O values with rainfall amounts because these values can also be influenced by various moisture sources, water circulation through underground networks, and the influence of climatic conditions on stalagmite formation (8, 11, 14, 26). Another qualitative precipitation proxy, δ^18^O of planktonic foraminifera, implies that the surface water salinity in the northern Bay of Bengal was slightly lower during the Holocene than during the Last Interglacial (27). This indicates that freshwater input from the catchment of the Ganges–Brahmaputra–Meghna (G-B-M) river system and ultimately ISM rainfall intensity during the Holocene might have been higher or at least similar despite higher insolation and higher SST in the tropical Indian Ocean during the Last Interglacial (27). Since salinity is not a direct measure of the rainfall amount but rather a proxy for freshwater runoff (28, 29), which could also be driven by mountain glacier melt, applying additional hydrological proxies at the same location can help to elucidate differences in ISM intensity between the two interglacial periods (8).

To test whether monsoon rainfall was indeed higher during the Last Interglacial than during the Holocene, we provide regional proxy records of ISM rainfall and vegetation changes in the G-B-M river catchment obtained from a marine sediment core from the northern Bay of Bengal that spans the last ∼130 kyr at submillennial-scale resolution for the Holocene and MIS 5e. This sediment core covers the last two interglacial periods and six precessional cycles, allowing us to scrutinize the relationship between insolation and rainfall intensity for the Bay of Bengal branch of the ISM. In particular, we establish records of the stable hydrogen and carbon isotope composition (δD and δ^13^C) of sedimentary leaf wax lipids, namely, long-chain *n*-alkanes, that have been extracted from the sediments. Studies of modern surface sediments in the northern Bay of Bengal and moisture source modeling, as well as a continuous ∼18-kyr-long marine sediment record, have confirmed that the δD and δ^13^C of long-chain *n*-alkanes are reliable proxies for ISM rainfall amount and vegetation changes in the G-B-M catchment (10, 30, 31). To complement our compound-specific stable isotope records, we compare these with published proxy data from the same sediment core (27) as well as with other Asian paleomonsoon proxy records.

## Geological Setting and Proxy Records

**Fig. 1.**
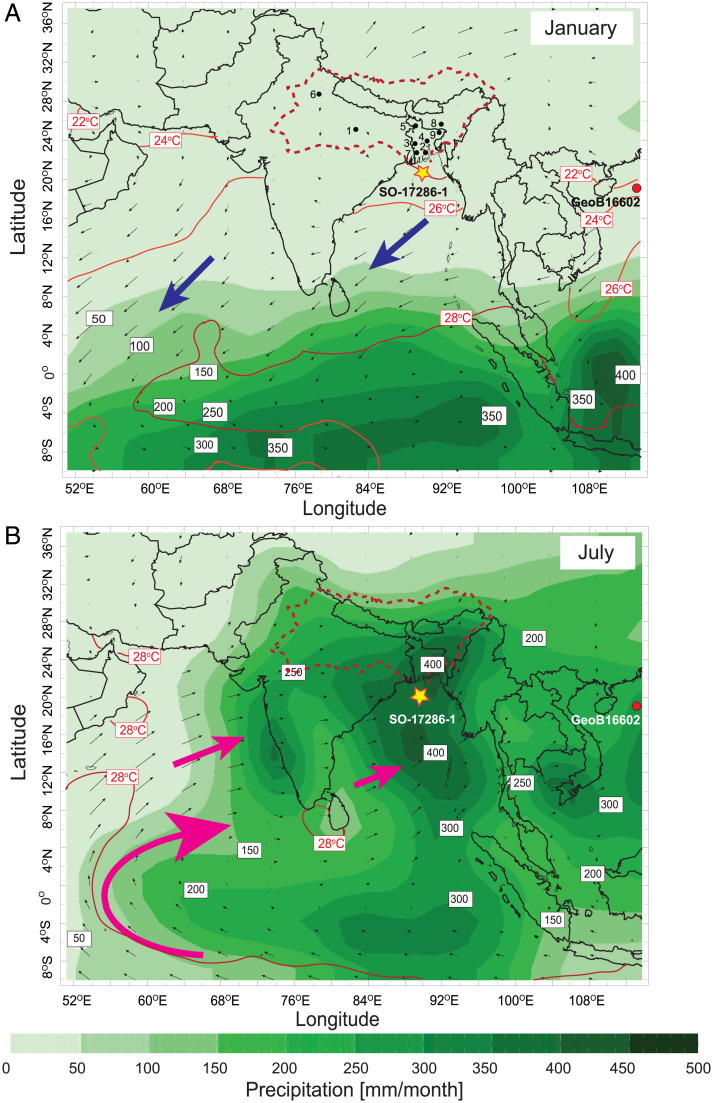
Modern rainfall variability over South Asia. SST (red; 2 °C isotherms), amount of precipitation (green; mm per month), and surface wind direction and strength (arrows with speed proportional to vector length) over the Indian Ocean, India, and Southeast Asia for (*A*) January and (*B*) July. The position of marine sediment core SO 188-17286-1 (this study) is indicated by a yellow star, and the G-B-M catchment is marked by a dashed red line. The position of marine sediment core GeoB16602 from the South China Sea (16) is indicated by a red dot. Large pink and blue arrows indicate monsoonal wind directions for the summer and winter seasons, respectively. Monthly SST and precipitation as well as surface wind speed and strength for the 925-hPa pressure level were derived from National Centers for Environmental Prediction reanalysis data (http://iridl.ldeo.columbia.edu). Black circles mark weather stations (1, Allahabad; 2, Barisal; 3, Chuadanga; 4, Dhaka (Savar); 5, Dinajpur; 6, New Delhi; 7, Satkhira; 8, Shillong; and 9, Sylhet) in the G-B-M catchment where the International Atomic Energy Agency has collected meteorological data (48).

The 984-cm-long marine sediment core SO 188-17286-1 (hereafter 17286-1) was retrieved in the northern Bay of Bengal from a site located at the continental slope off Bangladesh (19°44.58′N, 89°52.76′E, 1,428 m water depth), ∼220 km south of the mouth of the G-B-M river system (Fig. 1). The G-B-M river system drains an area of ∼1.7 × 10^6^ km^2^ and supplies ∼1,120 km^3^ of fresh water and ∼1,060 Mt of sediment per year from the Himalayas and the northern Indian subcontinent to the Indian Ocean (32). In terms of riverine sediment load it represents the largest single-entry point of sediment into the world’s oceans, providing the most dominant source of terrestrial material into the northern Bay of Bengal (30–32). Regional climate conditions are dominated by the summer monsoon; 70% of the annual precipitation occurs between June and September, while the winter monsoon season from October to May is subordinate with only 30% of the annual rainfall (Indian Institute of Tropical Meteorology; https://www.tropmet.res.in). This contrast is concomitant with the annual SST cycle and its associated heat and moisture advection (Fig. 1 *A* and *B*). During the summer monsoon season, the prevailing southwesterly winds bring moisture across the Indian Ocean to South Asia when the SST maximum is situated in the northern Indian Ocean. In contrast, the winter monsoon is dominated by less humid northeasterly winds as the SST maximum is situated farther south in the equatorial Indian Ocean. More than 95% of the sediments in the Bay of Bengal are transported from continental South Asia during the summer monsoon season (33).

Besides the massive sediment flux, the organic matter provided by the G-B-M river system is well preserved due to the extreme organic matter burial efficacy in the Bengal Fan (31, 34). Therefore, the sediments in the Bay of Bengal provide excellent hydrological and environmental archives at the basin scale. To investigate continental monsoon rainfall and vegetation changes in the G-B-M catchment, we determined the δD and δ^13^C values of terrestrial plant leaf wax lipids preserved in the sediments of core 17286-1. In monsoonal regions, changes in meteoric water δD are generally considered to reflect changes in the amount of rainfall with more negative δD values being indicative of higher rainfall amounts and vice versa (35, 36). Regional meteoric water δD is regarded as the primary control on the δD of the leaf wax lipids when the influence of changes in vegetation types or plant life forms is negligible (37–39). Thus, δD of terrestrial plant leaf waxes is commonly used as a rainfall proxy for large continental regions (10, 15, 39–41). In addition, changes in the δ^13^C of the long-chain *n*-alkanes can be used to track region-wide vegetation shifts (10, 15, 39–41) because C_3_ plants (i.e., rainforest and cool-season grasses and sedges) and C_4_ plants (i.e., warm-season grasses and sedges) significantly differ in their *n*-alkane δ^13^C values, ranging from −29 to −39‰ for C_3_ plants and from −14 to −27‰ for C_4_ plants (42–46).

The age model of sediment core 17286-1, which is described in detail elsewhere (27), is based on Bayesian age modeling using 12 radiocarbon dates in the upper ∼400 cm of the sediment core as well as 11 characteristic age markers established through correlation of the δ^18^O record of benthic foraminifera from this sediment core with the chronologically well-constrained LS16 global δ^18^O_benthic_ stack (47). According to the age model, sediment core 17286-1 spans the last ∼130 kyr with a fairly constant sedimentation rate of 5 to 10 cm kyr^−1^, except for a rapid short-term increase around the time of the Younger Dryas (YD; *SI Appendix*, Fig. S1). The average temporal resolution of the obtained compound-specific stable isotope data between 18.5 ka and present is 415 y, with highest resolution during the early Holocene (∼157 y) and the YD (∼70 y), while it is 561 y between 130 and 105 ka.

## Results

### Variations of *n*-Alkane δD and δ^13^C Values.

Higher terrestrial plants are the predominant plant contribution in the northern Bay of Bengal sediments as indicated by the carbon preference index (CPI) of the total long-chain *n*-alkanes (*n*-C_24_ to *n*-C_34_), which ranges between 3.5 and 6.5 with an average of 4.9 (*Methods*). The δD and δ^13^C values of the four dominant *n*-alkanes (*n*-C_27_, *n*-C_29_, *n*-C_31_, and *n*-C_33_) show similar trends over the last ∼130 kyr (*SI Appendix*, Fig. S2). After correcting the measured raw δD values for the effect of changes in continental ice volume on global seawater isotopes (39) (*Methods*), the resulting ice volume–corrected δD (δD_ivc_) values of the four dominant *n*-alkanes are on average ∼5.5‰ more negative than the raw values, but overall trends are still similar (*SI Appendix*, Fig. S3).

### δD_ivc_ Values in Sediment Core 17286-1 Reflect ISM Intensity and Rainfall Amount.

Multiple factors besides rainfall amount could have confounded the δD values of the leaf wax *n*-alkanes as a proxy for ISM intensity in the hinterland of the northern Bay of Bengal. For example, different vegetation types can bias interpretation of δD_ivc_ values because C_4_ grasses and C_3_ trees have a different apparent δD fractionation. However, the pairwise δD_ivc_ and δ^13^C values of each *n*-alkane homolog in our study show no correlation (all R^2^_adj_ ≤ 0.12; *SI Appendix*, Fig. S4), indicating that the effect of changes in the predominant vegetation functional types (C_4_ vs. C_3_ plants) and their photosynthetic pathways on δD is negligible (39). In addition, a correction for the temperature effect on the δD of precipitation in the G-B-M catchment is also not necessary as there is no correlation between modern precipitation δD and air temperature (R^2^_adj_ = 0.07; *SI Appendix*, Fig. S5) for all weather stations in the G-B-M catchment (*SI Appendix*, Fig. S6; meteorological data available in ref. 48). Therefore, we can rule out significant effects of vegetation type (C_4_ vs. C_3_ plants) and air temperature changes on the δD signal. Furthermore, although the headwaters of the Ganges and Brahmaputra rivers originate in the Himalayan mountain range, snow/ice melt accounts for less than 25% of the stream flow (49, 50), making rainwater the dominant water source for plants in the entire G-B-M catchment (49). Finally, unlike central India, where the δD of precipitation is strongly affected by moisture source changes between the western and central Indian Ocean and the Bay of Bengal as well as the Ganges Plain (12, 51), numerous studies using modern surface and down-core sediments as well as moisture source isotope modeling have suggested that the δD of precipitation in the G-B-M catchment predominantly reflects precipitation amount rather the changes in regional moisture sources (10, 30, 34, 52).

**Fig. 2.**
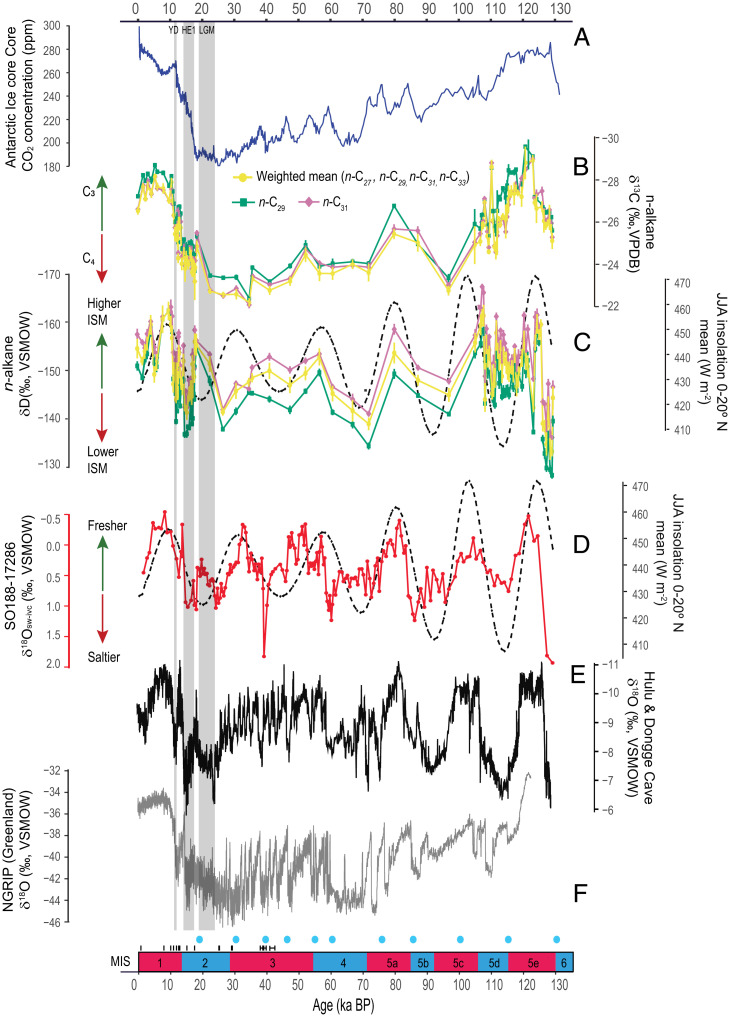
Climate records derived from sediment core 17286-1 in comparison with other high- and low-latitude climate proxy records: (*A*) Antarctic ice cores composite CO_2_ concentration (53, 54). (*B*) δ^13^C (mean ± SE) of *n*-C_29_ and *n*-C_31_ and concentration-weighted average δ^13^C based on all four homologs (*n*-C_27_, *n*-C_29_, *n*-C_31_, and *n*-C_33_) from sediment core 17286-1 as a proxy for vegetation (C_3_ vs. C_4_ plants) changes (this study). (*C*) Ice volume–corrected δD (mean ± SE) of *n*-C_29_ and *n*-C_31_ and concentration-weighted average δD_ivc_ based on all four homologs (*n*-C_27_, *n*-C_29_, *n*-C_31_, and *n*-C_33_) from sediment core 17286-1 as a proxy for rainfall amount (this study). The dashed black line represents summer (JJA) mean insolation at 0 to 20°N (55). (*D*) Ice volume–corrected planktonic foraminiferal δ^18^O (δ^18^O_sw-ivc_; red) as a proxy for local sea water salinity compared to boreal summer insolation as in Fig. 2*C* (27). (*E*) δ^18^O record from Chinese stalagmites (56), reflecting past EASM changes. (*F*) North Greenland Ice Core Project (NGRIP) ice core δ^18^O record (57), reflecting climate variability and particular Heinrich events in the Northern Atlantic realm. Gray bars mark the YD, HE1, and the LGM as reflected in the NGRIP ice core δ^18^O record as well as their correlatives in the Asian monsoon proxy records. Age control points for sediment core 17286-1 are also indicated, including radiocarbon dates (black lines) and tie points (blue circles) derived from tuning of the benthic foraminifera δ^18^O record to the LS16 global δ^18^O_benthic_ stack (47). Red and blue intervals at the bottom delineate MISs, separating warm and cold climate periods, respectively.

Out of the four long-chain *n*-alkanes under consideration (*SI Appendix*, Fig. S7), the most abundant homologs are *n*-C_29_ and *n*-C_31_. We therefore present the δD and δ^13^C values of these two *n*-alkanes to infer ISM rainfall and vegetation changes in the G-B-M catchment. These two dominant homologs likely derived from the same vegetation source because 1) changes in δ^13^C values for *n*-C_29_ and *n*-C_31_ closely track each other and 2) δD_ivc_ values of *n*-C_29_ and *n*-C_31_ are also strongly correlated (*P* < 0.001, *R*^2^ = 0.93, *n* = 99). To simplify the visualization, we also plotted the concentration-weighted δD_ivc_ and δ^13^C based on all four *n*-alkanes and their propagated errors (Fig. 2 *B* and *C*). Notably, the concentration-weighted δD_ivc_ and δ^13^C mean values closely mimic the changes in the δD_ivc_ and δ^13^C values of *n*-C_29_ and *n*-C_31_ (Fig. 2 *B* and *C*).

### ISM Rainfall Shifts in South Asia.

**Fig. 3.**
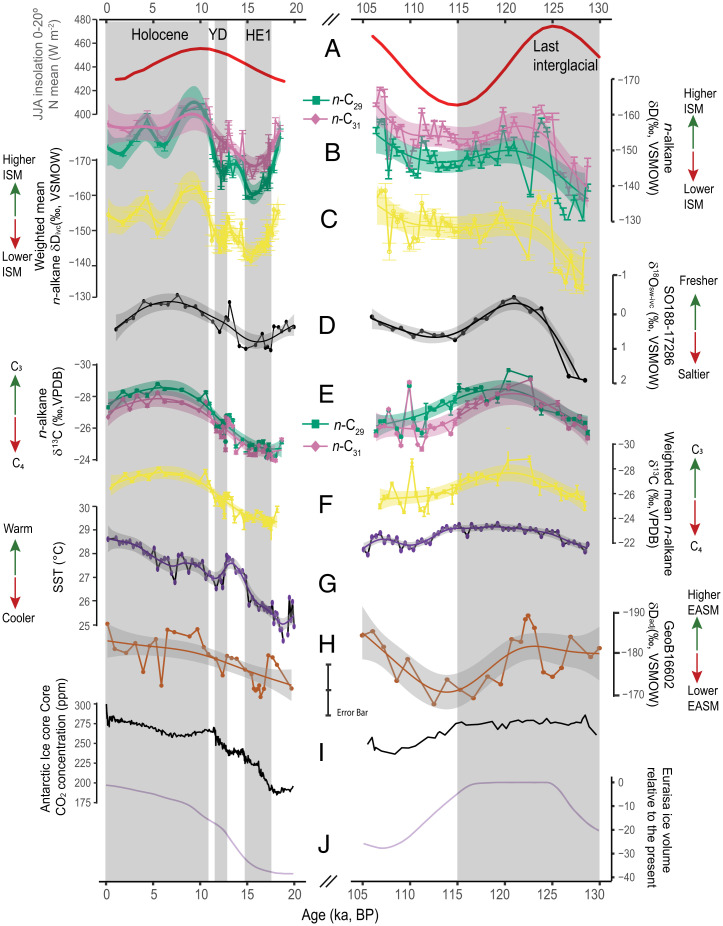
Detailed comparison of GAM results from paleoclimate proxy records in sediment core 17286-1 and climate forcing during the Holocene and the Last Interglacial. (*A*) Summer (JJA) mean insolation at 0 to 20°N (55). (*B*) δD_ivc_ of *n*-C_29_ and *n*-C_31_ and GAM results with 95% confidence intervals. (*C*) concentration-weighted δD_ivc_ based on all four homologs (*n*-C_27_, *n*-C_29_, *n*-C_31_, and *n*-C_33_) and respective GAM results with 95% confidence interval. (*D*) Freshwater runoff inferred from δ^18^O_sw-ivc_ (27). (*E*) Vegetation trends as inferred from *n*-alkane δ^13^C (mean ± SE) and GAM results for *n*-C_29_ and *n*-C_31_ with 95% confidence intervals. (*F*) Concentration-weighted δ^13^C based on all four homologs (*n*-C_27_, *n*-C_29_, *n*-C_31_, and *n*-C_33_) and GAM results with 95% confidence interval. (*G*) U^K’^_37_-based SST record of sediment core 17286-1 (27). (*H*) Amount-weighted mean *n*-alkane δD record (adjusted for ice volume and temperature effect) from South China Sea sediment core GeoB16602 (16). (*I*) Antarctic ice core composite CO_2_ concentration (54, 59). (*J*) Eurasia ice volume relative to present day (21). The gray bars mark the Holocene (11.6 ka to present) and the Last Interglacial (130 to 115 ka), as well as the YD and HE1.

Our records show that the δD_ivc_ values of *n*-C_29_ and *n*-C_31_ range between −128 and −170‰ over the last ∼130 kyr, revealing abrupt shifts between intervals of intensified and weakened ISM rainfall at the interglacial–glacial transitions. At the onset of the Holocene and the Last Interglacial, abrupt drops in δD_ivc_ of ∼25 to 28‰ indicate rapid and comparable increases in ISM intensity at the end of both glacial terminations (Fig. 2*C*). From the Last Glacial Maximum (LGM) to the Holocene, ISM intensity increased in two clear successional steps, which is a typical pattern in the Afro-Asian monsoon domain (28, 58). The weakest monsoon period (with the highest δD_ivc_ values) is concomitant with Heinrich event 1 (HE1) (18.0 to 14.8 ka) and was followed by a rainfall increase (∼12‰ decrease in δD_ivc_) until the onset of the Late Glacial interstadial (i.e., Bølling–Allerød chronozone) at 14.6 ka. Reduced rainfall characterized the interval equivalent to the YD (∼12.9 to 11.6 ka) before peak ISM rainfall intensity was reached during the early Holocene (11.6 to 9.8 ka) (Figs. 2*C* and 3*B*). At the onset of the Last Interglacial, δD_ivc_ values rapidly increased by 11‰ at 129 ka, reaching the weakest ISM intensity with the most positive δD_ivc_ values in our record. The maximum rainfall intensity was reached at 124.5 ka, followed by an ∼2,300-y-long increase in δD_ivc_ by 20‰.

Besides large shifts in ISM strength at the two glacial–interglacial transitions, our δD_ivc_ record also displays a large variability and abrupt changes in ISM rainfall throughout both the Holocene and the Last Interglacial. During the Holocene, the total δD_ivc_ range is 15‰, and the transition toward a weakened ISM during the middle and late Holocene is abrupt, occurring within ∼1,800 y. During the Last Interglacial, ISM intensity was even more variable with a δD_ivc_ range of 20‰ with an abrupt shift to a weaker ISM at 120 ka.

To gain a more comprehensive picture of past ISM variability, we compared the δD_ivc_-based rainfall intensity record with river runoff/freshwater input recorded by the δ^18^O of planktonic foraminifera in the same sediment core (27). The residual seawater δ^18^O (δ^18^O_sw-ivc_) values derived from the δ^18^O of the planktonic foraminifera (corrected for the effects of continental ice volume changes and calcification temperature on the δ^18^O of foraminifera shells) reflect isotopic changes in the Bay of Bengal surface water. Such isotopic changes are primarily driven by the variable contribution of ^18^O-depleted riverine freshwater discharge from the G-B-M river system (27). Our result shows that the δ^18^O_sw-ivc_ data closely correspond to shifts in δD_ivc_ in relative amplitude and timing, demonstrating a strong connection between continental precipitation and riverine fresh water runoff. That is, enhanced freshwater discharge from the G-B-M river system occurred during phases of intensified ISM and vice versa.

### Vegetation Changes in South Asia.

The δ^13^C values of *n*-C_29_ and *n*-C_31_ as well as the concentration-weighted average span 8‰ (−22 to −30‰). The highest δ^13^C values (on average −22‰), indicating a prevalence of C_4_ vegetation, occurred from ∼36 to 21 ka, a period encompassing the onset of global ice sheet growth and the LGM. Similar to δD_ivc_, an abrupt decrease in δ^13^C by ∼4‰ occurred at the onset of both Terminations I and II (Fig. 2*B*), reflecting increases in the proportion of C_3_ vegetation. There is also a clear two-step C_3_ vegetation expansion at the transition from the last deglaciation to the Holocene, mimicking the two-step increase in monsoon rainfall inferred from the δD_ivc_ record. The lowest δ^13^C values (on average −29‰) occurred during both the early to middle Holocene and the Last Interglacial, showing a similar dominance of C_3_ vegetation in the G-B-M catchment during both intervals (Fig. 3*E*). The overall δ^13^C trend generally follows the δD_ivc_ shifts throughout the last ∼130 kyr, suggesting that the waxing and waning of monsoon intensity during interglacial/interstadial and glacial/stadial climate states was accompanied by significant changes in vegetation types (Fig. 2 *B* and *C*). However, there are also periods of asynchronous changes in δ^13^C and δD_ivc_, particularly during MIS 5e and MIS 5d as well as during the middle and late Holocene. This decoupling shows that vegetation changes do not always follow the rainfall changes but rather the atmospheric CO_2_ concentration (Fig. 2 *A* and *B*) because elevated CO_2_ levels generally favor C_3_ over C_4_ plants (60). Considering similar C_3_ plant type cover during both interglacial periods implies that vegetation types do not bias our interpretation of the δD_ivc_ values for two reasons: 1) similar vegetation types would have similar apparent δD fractionation between rainfall and leaf wax (39) and 2) any vegetation effect on water isotope values (i.e., δ^18^O and δD) through changes of temperature and soil wetness was similar (61).

## Discussion

### Climatic Controls on ISM Intensity at Millennial to Orbital Time Scales.

The long-term hydroclimate evolution, recorded by the concentration-weighted δD_ivc_-inferred rainfall amount and the δ^18^O_sw-ivc_-inferred freshwater runoff, largely corresponds to changes in mean summer (June, July, and August [JJA]) insolation at 0 to 20°N, i.e., over the northern tropical Indian Ocean, throughout the last ∼130 kyr (Fig. 2 *C* and *D*). The sediment core 17286-1 ISM intensity record is virtually in phase with local summer insolation at the precessional band, i.e., at 19 to 23 kyr cycles, corroborating stalagmite δ^18^O records from northern India and southwest China as well as foraminifera δ^18^O data from the northern Bay of Bengal (Fig. 2*D*) (9, 11, 27). The hydrological records of sediment core 17286-1 are also remarkably similar in terms of amplitude and timing to changes in the East Asian summer monsoon (EASM) recorded by stalagmite δ^18^O from Hulu and Dongge caves in eastern China during the last ∼130 kyr (56) (Fig. 2*E*), supporting the importance of insolation forcing on both monsoon systems at orbital scales. Furthermore, during the last glacial termination, the overall increase in monsoon intensity observed in sediment core 17286-1 was also punctuated by abrupt weak ISM events (i.e., during HE1 and the YD), demonstrating that climatic changes in the North Atlantic realm exert an important control on ISM intensity at centennial to millennial time scales (Fig. 2*F*). This is consistent with previously documented links between weak ISM intervals inferred from δ^18^O_sw-ivc_ data from the same sediment core and North Atlantic Heinrich events (Fig. 2*D*) (27).

### Internal Climatic Feedback of South Asian Monsoon Variability during the Last Interglacial and the Holocene.

The sediment core 17286-1 proxy records clearly show that ISM rainfall shifts are paced by summer insolation and North Atlantic climate variability over the last ∼130 kyr. However, from the comparison of the peak δD_ivc_ values for the early Holocene (−162 and −163‰ for *n*-C_29_ and *n*-C_31_, respectively) and the early Last Interglacial (−158 and −162‰ for *n*-C_29_ and *n*-C_31_, respectively) it appears that monsoon strength was not lower, as expected by insolation, but instead higher during the early Holocene than during the early Last Interglacial (Fig. 3*B*). To ensure the robustness of these single-point inferences, we derived broader trends for rainfall intensity using a generalized additive model (GAM) for both interglacial periods (the parametric coefficients and significances of smooth terms are shown in *SI Appendix*, Table S1). As a result, the GAMs demonstrate with 95% confidence that δD_ivc_ values were higher during the early Holocene than during the early Last Interglacial (Fig. 3 *B* and *C*). Indeed, the respective mean δD_ivc_ values of *n*-C_29_ (−154‰) and *n*-C_31_ (−157‰) during the Holocene are 10 and 6‰ higher than their counterparts during the Last Interglacial (*n*-C_29_ = −144‰; *n*-C_31_ = −151‰). By applying a multivariate analysis of variance (MANOVA), we find that the δD_ivc_ values of both interglacial periods are distinct (Pillai’s trace = 0.3, *F*_(2,30)_ = 5.7, *P* = 0.008; *SI Appendix*, Table S2), suggesting that ISM intensity during the Last Interglacial was lower than during the Holocene. This is further supported by the riverine freshwater runoff record from the same sediment core, revealing a peak δ^18^O_sw‐ivc_ value of −0.54‰ during the Holocene compared to −0.47‰ during the Last Interglacial. GAMs of river runoff (δ^18^O_sw‐ivc_) during both periods show that these values are not significantly different (one-way ANOVA, *F*_(1,20)_=2.75, *P* = 0.113; *SI Appendix*, Table S3 and Fig. 3*D*).

To compare δD_ivc_ values and infer rainfall amounts during different warm climate states, we also examined whether the relationship between δD_ivc_ and rainfall amount was constant during the Holocene and the Last Interglacial. Several isotope-enabled climate model simulations for these two interglacials have shown that water isotope values such as δ^18^O and δD are strongly inversely correlated with the amount of precipitation in the ISM region at different interglacial stages (62–64) (*SI Appendix*, *Supplementary Background 1*). In transient water isotope simulations with the fully coupled Max Planck Institute for Meteorology (MPI-M) atmosphere (ECHAM5) and ocean (MPI Ocean Model [MPI-OM-1]) models, hereafter as COSMOS-wiso model (65), this inverse correlation between δD and the precipitation appears to be mostly stable over time in the G-B-M catchment. For simulations of the Last Interglacial (130 to 115 ka) (65) and the Holocene (7 to 0 ka) (66), we find the ratio between simulated δD and rainfall amount to be stable at orbital time scales except for the first 4,000 y of the interglacial period (*SI Appendix*, Fig. S8). During the early part of the Last Interglacial the two variables appear to be decoupled with simulated precipitation gradually decreasing while δD does not display any substantial change. This decoupling could hint at an underestimated rainfall amount in the early part of Last Interglacial, but this could largely be due to incomplete parameterization of the mechanisms controlling rainfall (*SI Appendix*, *Supplementary Background 1*). The early period of an interglacial might still be affected by the glacial–interglacial transition, which, by design, is not represented in the model setup (65). Once the transient model enters a stable interglacial state (e.g., at 126 and 7 ka), δD and precipitation amount converge and are significantly inversely correlated, demonstrating similar δD ranges corresponding to similar precipitation estimates (*SI Appendix*, Fig. S8). It is noteworthy that during the Last Interglacial (130 to 115 ka), both proxy and modeled δD data show similar trends with the most negative δD values (e.g., leaf wax and modeled precipitation) occurring at ∼125 ka. However, the leaf wax δD change rate from 130 to 126 ka in our record is much greater than that in the simulated rainfall δD (Fig. 3*B* and *SI Appendix*, Fig. S8*B*). The observed weak relation in the COSMOS transient simulation from 130 to 126 ka does not affect our interpretation of δD peaks at 124.5 ka because the relationship of δD and rainfall amount is very tight between 126 and 115 ka. In addition, the highest leaf wax δD peak at 124.5 ka is comparable to that at 7 ka during the Holocene, but still lower than the Holocene δD peak at 9 to 11 ka (Fig. 3*B*). Taken at face value, this implies that the precipitation at the peak of Last Interglacial should be similar to that at 7 ka. This further suggests that precipitation in MIS 5e was not higher despite the higher insolation. Taken together, δD_ivc_ data are a robust proxy for quantifying ISM rainfall during the Last Interglacial and Holocene (*SI Appendix*, *Supplementary Discussion 1*).

Therefore, our finding challenges results from several fully coupled atmosphere–ocean general circulation model (AOGCM) simulations, which predict higher ISM intensity during the Last Interglacial than during the Holocene (17, 18). According to these models, higher prescribed insolation leads to higher precipitation during the early Last Interglacial (around 126 ka) than during the early Holocene (around 9.5 ka). On the contrary, our data from sediment core 17286-1 clearly indicate that insolation is not the sole determining factor for ISM intensity, suggesting that other factors may also have critically influenced rainfall variability during both interglacial periods (7, 8, 17). The discrepancy between the sediment core 17286-1 proxy data and AOGCM simulation results highlights the importance of paleomonsoon proxy studies to improve climate models and to distinguish between different natural forcings of monsoon rainfall and their interaction at different time scales (7, 8, 17, 67).

One plausible control to dampen the ISM intensity during the Last Interglacial thermal maximum could be due to Indian Ocean warming and a changed distribution of rainfall between land and ocean. Although the effects of SST changes on ISM intensity are still under debate (18, 68), several paleoclimate model studies proposed that an SST increase in the Indian Ocean would reduce South Asian monsoon intensity on land because higher SST during summer favors local convection over the ocean (4, 69, 70). This enhanced convection over the ocean is compensated by subsidence of air over the continent at 10 to 20°N, inhibiting moist air convection over the land mass and drying the region through a modulation of the local Hadley cell (2, 70). Indeed, data extracted from transient climate simulations with the COSMOS-wiso model show that modeled rainfall in the G-B-M catchment is anticorrelated with modeled convective precipitation (*SI Appendix*, Fig. S9*A*) and modeled SSTs (*SI Appendix*, Fig. S9*B*) in the northern Bay of Bengal during both interglacials (65, 66). These model results strongly suggest that higher SSTs in the northern Bay of Bengal can exert a dampening effect on land precipitation in the ISM region.

**Fig. 4.**
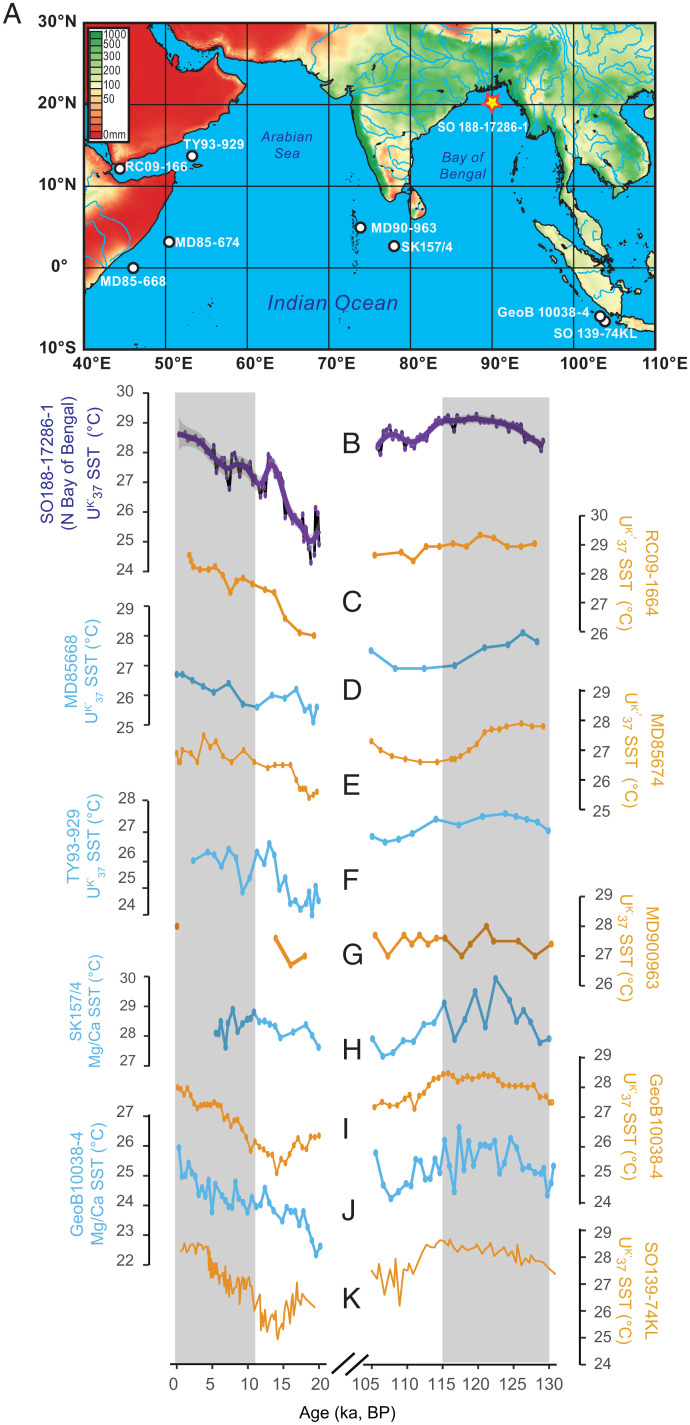
Comparison of SST records from the equatorial and tropical Indian Ocean during the Holocene and the Last Interglacial. (*A*) Map of the ISM domain with average July precipitation [WorldClim1 30 arc sec gridded precipitation data (71)] and the locations of sediment core 17286 (yellow star) and other regional SST records (white dots) that are displayed below. (*B*) U^K’^_37_-based SST record of sediment core 17286-1 (27). Marine sediment SST records based on either U^K’^_37_ or *G. ruber* Mg/Ca from tropical or equatorial Indian Ocean arranged in a west to east sequence: (*C*) U^K’^_37_-based SST record of sediment core RC09-1664 (72). (*D*) U^K’^_37_-based SST record of sediment core MD85668 (73). (*E*) U^K’^_37_-based SST record of sediment core MD86674 (73). (*F*) U^K’^_37_-based SST record of sediment core TY93-929 (74). (*G*) U^K’^_37_-based SST record of sediment core MD900963 (75). (*H*) *G. ruber* Mg/Ca-based SST record of sediment core SK157/4 (76). (*I*) U^K’^_37_-based SST record of sediment core GeoB10038-4 (77). (*J*) *G. ruber* Mg/Ca-based SST record of sediment core GeoB10038-4 (77). (*K*) U^K’^_37_-based SST records of sediment core SO139-74KL (78).

Therefore, we argue that Indian Ocean warming during the Last Interglacial may explain the muted ISM intensity at that time within the G-B-M catchment. Indeed, the alkenone unsaturation index (U^K’^_37_)-based SST record from our core location (27) shows that average SSTs during the peak Last Interglacial (∼125 ka) were 1.5 to 2.5 °C higher than during the early Holocene (Figs. 3*G* and 4*B*). In fact, warmer SST apparently prevailed during the Last Interglacial across the equatorial and northern Indian Ocean. This is evident from compiled SST records based on the two commonly used paleothermometers U^K’^_37_ and *Globigerinoides ruber* Mg/Ca (Fig. 4 *A*, *C*–*K*). A recent study using both model simulations and observational data from 1901 to 2012 also demonstrated that ISM intensity has weakened in South Asia, including a substantial portion of the G-B-M catchment, as a result of sustained high SSTs (>28 °C) in the western Indian Ocean since the 1950s (2). Although the recent Indian Ocean warming may be largely attributed to anthropogenic rather than to natural forcing (79), it is obvious that SST changes played an important role in modulating rainfall on land (2).

Although we did not obtain a high-resolution rainfall proxy record for entire MIS 5c (*SI Appendix*, *Supplementary Discussion 2*), the concentration-weighted δD_ivc_ record infers higher rainfall at the beginning of MIS 5c (∼106 ka) than during the peak Last Interglacial (∼125 ka) despite almost identical insolation during these two periods (Fig. 3*C*). Again, this could be explained by lower Indian Ocean SSTs (up to 0.5 °C) during MIS 5c than during MIS 5e (Fig. 3*G*), resulting in lower convectional rainfall above the ocean but higher ISM intensity on land at the beginning of MIS 5c. Likewise, we observed slightly higher ISM intensity (GAM-modeled concentration-weighted δD_ivc_) during the early Holocene than during MIS 5c, which could also be due to lower SSTs (by up to 1 °C) in the western Indian Ocean during the Holocene (Fig. 3 *C* and *G*). Although we propose that SST dampens ISM intensity during the interglacial states, this by no means suggests that ISM intensity was highest when SST was lowest because this is obviously not the case for glacial/interglacial transitions as well as for glacial periods (Fig. 2*C*). Our study demonstrates the needs for improving parametrization of the ISM control mechanisms for both interglacial and glacial climate states as well as for the transition between these drastic climate states as the relationship between insolation and rainfall is also not constant. Taken together, this underscores the importance of better understanding monsoon rainfall variability during warmer-than-Holocene interglacial climate states, e.g., during MIS 5e.

Other possible mechanisms affecting ISM intensity could be the influence of GHG concentrations (8, 12) and vegetation cover changes (8). Proxy evidence as well as model simulations suggest that GHG concentrations have played an important role in developing wet interglacial conditions in Africa’s monsoonal regions (80) and the ISM core region in central India (12). However, we rule out a direct GHG effect on ISM intensity in the G-B-M catchment because the global CO_2_ concentration was higher during the early Last Interglacial than during the early Holocene (53, 54) (Figs. 2*A* and 3*G*). Moreover, when the CO_2_ concentration continued to increase during the early Holocene (Fig. 3*I*), ISM intensity decreased after the early Holocene peak, a feature mainly reflecting changes in insolation (Fig. 3 *A* and *C*). Forcing and feedback associated with changes in vegetation cover may also have critical effects on monsoon systems (8). The smaller Eurasian ice sheet volume (Fig. 3*J*) during the early Holocene compared to the Last Interglacial could have also affected the moisture and energy budgets of the South Asian monsoon system (21). For example, positive vegetation feedback in terms of expanded forest cover in Eurasia during the early and middle Holocene has been proposed as an amplifier of North African monsoon rainfall (81). However, as we stated in the results, C_3_ vegetation cover in the G-B-M catchment was similarly dominant during the Holocene and the Last Interglacial (Fig. 3 *E* and *F* and *SI Appendix*, Table S1), suggesting that the vegetation feedbacks on ISM intensity should have been similar for the two interglacial periods.

Notably, by visual inspection, we observe a small (∼1 to 2 kyr) time lag in precipitation maxima relative to summer insolation maxima in the Bay of Bengal proxy records. If the GAM curves of rainfall records are considered, the precipitation maxima for the G-B-M catchment are slightly delayed with respect to insolation maxima (Fig. 3 *B* and *C*). However, one could also argue that this delay is artificially caused by the superimposed millennial-scale drops in monsoon intensity. Occurring just before maximum monsoon intensity is reached, they probably mask a direct response of ISM intensity to precessional insolation changes. This time lag could also be related to the uncertainty of the sediment core chronology (27). Nonetheless, our work contrasts the strong phase lag of 8 to 11 kyr observed in the northern Arabian Sea, with precipitation maxima occurring clearly after isolation maxima, a lag supposedly caused by Southern Hemisphere climate variability (82, 83). However, aligned with the evidence from stalagmite δ^18^O data from northern India and proxy data from marine sediment cores from the Bay of Bengal, our records further confirm that Southern Hemisphere climate variability did not play a dominant role for ISM variability at millennial to orbital time scales (9–11, 27). The difference observed for the two branches of the ISM over the Bay of Bengal and the northern Arabian Sea implies that the underlying mechanisms and climate internal feedbacks that modulate the monsoon in these two regions are largely different.

It is unclear whether muted monsoon intensity during the Last Interglacial in the Bay of Bengal branch of the South Asian monsoon is a unique regional feature or a general global pattern. Additional δD studies in other tropic monsoon domains are needed to elucidate varying monsoon responses during the two interglacial periods. However, we caution against direct interregional comparisons of δD records obtained from terrestrial plant leaf waxes owing to different effects of rainfall amount, vegetation types, temperature, and varying mixing ratios of moisture sources (10, 12, 16, 39). For example, a δD*_n_*_-alkane_ record from the South China Sea shows, after adjusting for global ice volume change and temperature effects, an identical data range for the Holocene and the Last Interglacial (ANOVA; *F*_(1,29)_ =1.32, *P* = 0.26; Fig. 3*H*) with mean δD_adjusted_ values of −182 and −180‰, respectively (16). Yet, this δD*_n_*_-alkane_ record cannot be interpreted in terms of an absent difference in EASM intensity during the two interglacial periods because the δD values during the two interglacial periods were influenced by changes between Pacific-sourced and Indian Ocean–sourced moisture as well as by isotopically enriched precipitation from southeast China (16).

In addition, the use of different qualitative precipitation proxies (e.g., stalagmite δ^18^O, plant leaf wax δD, river runoff, and plant community changes) for reconstructing monsoon intensity complicates direct regional comparisons of monsoon intensity and variability during the Last Interglacial and the Holocene (11, 14, 15, 56, 84, 85). Given that these rainfall proxies reflect multiple influences and various aspects of changes in monsoon rainfall (7), inaccurate conclusions can be drawn when only one type of proxy is considered to simply reflect rainfall amount. For instance, one of the most outstanding issues in paleohydrological studies relates to understanding how various factors affect stalagmite δ^18^O records (8, 11, 14, 26). Thus, considering multiple monsoon rainfall proxies besides stalagmite δ^18^O is a prerequisite for deriving robust inferences of monsoon rainfall intensity controls and understanding the various aspects of changes in monsoon rainfall (10, 16, 30).

### Mechanisms Controlling Vegetation Variability in South Asia.

The compound-specific stable isotope records from sediment core 17286-1 show that vegetation shifts generally follow ISM intensity changes. Given that C_4_ vegetation is more drought tolerant compared to C_3_ vegetation, parallel shifts in vegetation types (derived from *n*-alkane δ^13^C) and ISM intensity suggest that the rainfall amount exerts an important control on vegetation changes in the G-B-M catchment (Fig. 2*B*). This is particularly evident for the last deglaciation, when the vegetation shifts were mimicking the ISM intensity increase in two clear successional steps before reaching a C_3_ vegetation peak during the early Holocene. This stepwise pattern corroborates a previous vegetation and ISM reconstruction for the G-B-M catchment during the last ∼18 kyr (10). However, there are also periods of asynchronous changes in vegetation and precipitation throughout the last ∼130 kyr, particularly during MIS 5e, MIS 5d, and the middle and late Holocene. For example, from 127 to 105 ka, ISM intensity was fairly variable (e.g., large δD shifts), whereas the vegetation, according to the more negative *n*-alkane δ^13^C signal, was relatively stable and dominated by C_3_ plants. This decoupling shows that vegetation changes do not follow the rainfall changes but rather follow changes in atmospheric CO_2_ concentration (Fig. 2 *A* and *B*). As elevated CO_2_ concentrations generally favor C_3_ over C_4_ plants (54), the stable dominance of C_3_ plants during both interglacials suggests that rainfall plays a less dominant role in regulating C_3_ and C_4_ vegetation shifts than CO_2_.

The effect of CO_2_ on vegetation was also evident during the Last Glacial (∼105 to 21 ka) when C_4_ vegetation was dominant in the G-B-M catchment with only small fluctuations (e.g., range of δ^13^C values <4‰) (Fig. 2*B*). Even when the ISM abruptly intensified around 105 ka, C_3_ vegetation continued to decline, following the decrease in CO_2_ concentration (Fig. 2*A*). In addition to precipitation and atmospheric CO_2_ concentration, growth temperature and the interaction among these three factors may have been important, together modulating changes in vegetation (86). Vegetation modeling studies are therefore warranted to elucidate how the interplay of these factors could have driven vegetation changes in the G-B-M catchment during different time periods.

### Perspectives.

The finding of somewhat muted ISM intensity in South Asia during the Last Interglacial points to the need for reevaluating the effect of insolation forcing on rainfall intensity in fully coupled AOGCMs. Although insolation was largely responsible for the precessional ISM pacing at glacial and interglacial cycles, our study reveals that insolation is not the only control of monsoon intensity. Growing evidence supports that a complex interaction of control mechanisms and internal climate feedbacks affected rainfall variability during interglacial periods (8, 16, 87). The dampened ISM response during the Last Interglacial was likely caused by relatively high Indian Ocean SSTs, which may have enhanced the convective power over the ocean and consequently decreased rainfall over the Indian subcontinent. This implies that Indian Ocean warming under the modern climate scenario may continue to induce severe dry monsoon extremes over a big portion of the Indian subcontinent, which is in agreement with recent observations (2, 88).

Understanding the controls of monsoon systems is vital for adapting societies to the extremes between drought and floods that impact farmland, natural ecosystems, and consequently the livelihood of billions of people. The discrepancy between proxy data and model simulations highlights that hydroclimate proxy records are pivotal for understanding the potential range and rate of climate change. The abrupt ISM rainfall changes documented for the two interglacial periods provide new input for climate models to isolate different mechanisms and test model-derived hypotheses. More multiproxy paleorecords from different monsoon regions that compare natural warm climate states are therefore needed to aid predicting the impacts of future global warming and developing adaptation strategies.

## Methods

### Age Model.

The age model for sediment core 17286-1 (27) was developed by Bayesian age modeling using a P_Sequence depositional model (with variable parameter k and outlier analysis) implemented in OxCal 4.2.4 (89, 90) with the Marine13 calibration curve (91). As input parameters for the model we used accelerator mass spectrometry ^14^C dates of 12 samples of mixed planktonic foraminifera from the uppermost ∼400 cm of the sediment core (with a marine reservoir correction of 322 ^14^C yr (*R* = 400 y, ΔR = −78 ± 53 y) (92) as well as the ages of 11 characteristic stratigraphic tie points inferred from the correlation of the δ^18^O record of benthic foraminifera with the chronologically well-constrained LS16 global δ^18^O_benthic_ stack (47)

### *n*-Alkane Extraction and Analysis.

Bulk sediment samples from sediment core 17286-1 were extracted for total lipids according to the method described by Wang et al. (2013) with a slight modification regarding elemental sulfur removal (39). To obtain high-resolution records for the interglacial periods, we sampled at 2-cm intervals from 130 to 105 ka and then again from 19 ka to present. From 105 to 19 ka, we sampled at 30- to 50-cm intervals as our main goal was to compare the two interglacials. Approximately 4 to 8 g of homogenized dry sediment were extracted for total lipids (TLE) using a Dionex ASE 200 accelerated solvent extractor with a mixture of dichloromethane and methanol (DCM:MeOH, 9:1 v:v). After removing elemental sulfur by stirring the TLE with HCl-activated copper beads in DCM on a shaking table overnight, the saturated hydrocarbon fraction containing the *n-*alkanes was separated from the TLE by using column chromatography with activated silica gel (4 h at 450 °C) and hexane as eluent. To remove coeluting alkenes, the saturated hydrocarbon fraction was furthermore eluted with hexane over a column with AgNO_3_-impregnated silica gel. Identification of the individual compounds was carried out with an Agilent 6890 gas chromatograph (GC) with a flame ionization detector based on comparison with an in-house standard mixture of different *n*-alkanes (*n-*C_20_ to *n-*C_40_). Quantification of the *n*-alkane content was carried out using the same in-house *n*-alkane mixture (five-point calibration curves) for each compound. The CPI was calculated to examine the odd-over-even carbon number predominance to distinguish terrestrial plant from petroleum sources (93). The CPI is defined as

[1] $$\text{CPI}=\frac{\sum (X_{i}+X_{i+1}+..+X_{n})+\sum \left(X_{i+2}+..+X_{n}+2\right)}{2\times \sum \left(X_{i+1}+..+X_{n+1}\right)},$$

with *i* = 25 and *n* = 33. *X_i_* to *X_n_* refer to the abundances of the respective *n-*alkanes.

The *n*-alkane fraction was then analyzed at the Leibniz Laboratory for Radiometric Dating and Stable Isotope Research in Kiel for δ^13^C and δD using an Agilent 6890 GC, equipped with a CTC Analytics GC PAL autosampler, a Gerstel KAS 4 PTV injector, and an Agilent HP-5 capillary column (30 m × 0.32 mm × 0.25 µm), which was coupled to a Thermo Finnigan MAT 253 isotope ratio mass spectrometer via a Thermo Finnigan GC Combustion III interface (with pyrolysis oven and high-temperature combustion unit for δD and δ^13^C analyses, respectively).

When possible, each of the 92 samples was injected three to four times for *n*-alkane δ^13^C analysis. Measured δ^13^C values were calibrated against the external A4 *n*-alkane reference mixture (A. Schimmelmann, University of Indiana, Bloomington, IN) and are reported in the standard delta (δ) notation in per mil relative to the Vienna Pee Dee Belemnite (VPDB) standard. The accuracy and precision of the system were determined daily by measuring an internal *n*-alkane standard mixture (*n-*C_20_ to *n-*C_40_) between samples. The SEM for δ^13^C compared to the internal standard (on average 5 standards per day) was ≤0.13‰ for all compounds.

For *n*-alkane δD analyses (*n* = 102, on average four to six injections per sample), pyrolytic conversion of organic hydrogen to H_2_ was conducted at 1,450 °C. The H_3_^+^ factor was determined daily using H_2_ reference gas and varied between 5.37 and 5.98 ppm nA^−1^ over the analysis period of 4 mo, averaging at 5.66 ppm nA^−1^ with an SD of 0.16 ppm nA^−1^. δD values of the internal *n*-alkane standard mixture (*n-*C_20_ to *n-*C_40_) were calculated relative to pulses of reference H_2_ gas and then calibrated against the Vienna Standard Mean Ocean Water (VSMOW) scale by reference to H_2_ produced from the coinjected A4 *n*-alkane reference mixture. We then normalized the δD data to the VSMOW scale by using linear regression to the internal *n*-alkane standard mixture (*n-*C_20_ to *n-*C_40_), which was run daily with the samples. The SE for δD of the internal *n*-alkane standard (on average eight standards per day) was <1.0‰ for all compounds. All δD values are expressed in the standard delta (δ) notation in per mil relative to the VSMOW standard.

The obtained δD values were corrected for changes in the isotopic composition of global seawater caused by changes in continental ice volume following equations from Wang et al. (39). The simulated δ^18^O_ice-volume_ data were published by Bintanja et al. (21). The ice volume correction for the δD values is based on Jouzel et al. (94):

[2] $$\text{δD}={\text{δD}}_{\text{measured}}-8{\text{Δδ}}^{18}{\text{O}}_{\text{ice-volume}}\times \left(1+\frac{{\text{δD}}_{\text{measured}}}{1,000}\right)/\left(1+8\frac{{\text{Δδ}}^{18}{\text{O}}_{\text{ice-volume}}}{1,000}\right),$$

where δ^18^O_ice-volume_ is the ice volume contribution to changes in oceanic δ^18^O. The δD values discussed in this work are all referred to ice volume–corrected values (δD_ivc_).

### GAM on Paleoclimate Proxy Data.

To gain better insights into the broader hydroclimate and vegetation trends recorded in the sediment core 17286-1 proxy data during the Holocene and Last Interglacial, we ran GAM models in R (version 4.0.1, R-Development Core Team, 30 November 2017) with R Studio interface version 1.3.959 for each proxy dataset. The GAM allows us to fit our data into a more complex and nonlinear model, allowing a better prediction of the trends. The GAM smooth terms such as basis function (k) and smoothing parameter (λ) for each proxy dataset are listed in *SI Appendix*, Table S1. The models were checked for overfitting. The 95% confidence intervals for each proxy are plotted. To test the null hypothesis that there is no significant δD_ivc_ difference between the Holocene and the Last Interglacial, we applied Pillai’s trace MANOVA for these two interglacial periods. Then we also used univariate ANOVA performed on the output from MANOVA to assess which *n*-alkane is the most representative for separating the two periods. We also applied one-way ANOVA to test whether there is a significant difference in δ^18^O_sw‐ivc_ between the two interglacial periods. Unless otherwise stated, statistical significance is assessed at *P* < 0.05.

## Supplementary Material

Supplementary File

Supplementary File

## Data Availability

All *n*-alkane from sediment core SO 188-17286-1 are available in Dataset S1.
