# Supplementary material for: Higher sea surface temperature in the Indian Ocean during the Last Interglacial weakened the South Asian monsoon
Source: Proc Natl Acad Sci U S A. 2022 Mar 1;119(10):e2107720119. doi: 10.1073/pnas.2107720119 (PMC8915836; doi:10.1073/pnas.2107720119)
Supplement: Supplementary File [file pnas.2107720119.sapp.pdf]

Supplementary Material

Higher sea surface temperature in the Indian Ocean during the Last Interglacial weakened the South Asian monsoon

Yiming V. Wang[1*], Thomas Larsen[1], Stefan Lauterbach[2,3], Nils Andersen[2], Thomas Blanz[3], Uta Krebs-Kanzow[4], Paul Gierz[4], Ralph R. Schneider[2,3]

Affiliations:

1. Department of Archaeology, Max Planck Institute for the Science of Human History, 07745 Jena, Germany

2. Leibniz Laboratory for Radiometric Dating and Stable Isotope Research, Kiel University, 24118 Kiel, Germany

3. Institute of Geosciences, Kiel University, 24118 Kiel, Germany

4. Helmoltz Center for Polar and Marine Research, Alfred Wegener Institute, 27578 Bremerhaven, Germany

*Corresponding Author: Yiming V. Wang

E-mail: ywang@shh.mpg.de

**Supplementary Table S1**	Model parameters (basis function and smoothing parameter) for the Generalized Additive Model (GAM) for different proxies.
**Supplementary Table S2**	MANOVA and ANOVA results comparing average δD_{ivc} values for the Holocene and the Last Interglacial.
**Supplementary Table S3**	ANOVA test comparing the $\delta^{18}O_{sw\text{-}ivc}$ values for the Holocene and the Last Interglacial.
**Supplementary Fig. S1**	Sedimentation rate for sediment core SO 188-17286-1 over the last ~130 kyr.
**Supplementary Fig. S2**	Uncorrected δD and $\delta^{13}C$ values for the four dominant long-chain *n*-alkanes *n*-C_{27}, *n*-C_{29}, *n*-C_{31} and *n*-C_{33} from sediment core SO 188-17286-1.
**Supplementary Fig. S3**	Ice-volume-corrected δD (δD_{ivc}) values for the four dominant long-chain *n*-alkanes *n*-C_{27}, *n*-C_{29}, *n*-C_{31} and *n*-C_{33} from sediment core SO 188-17286-1.
**Supplementary Fig. S4**	Correlation between paired δD_{ivc} and $\delta^{13}C$ values for the four *n*-alkanes *n*-C_{27}, *n*-C_{29}, *n*-C_{31} and *n*-C_{33}. The lack of correlation between δD_{ivc} and $\delta^{13}C$ for each *n*-alkane homologue suggests a limited influence of vegetation type changes (C_4 vs. C_3 plants) on δD_{ivc}.
**Supplementary Fig. S5**	Correlation between precipitation δD and air temperature for IAEA weather stations in the G-B-M catchment.
**Supplementary Fig. S6**	Monthly mean rainfall (grey bars), air temperature (blue curves) and precipitation δD values (orange curves) for different weather stations in the G-B-M catchment (International Atomic Energy Agency, 2021).
**Supplementary Fig. S7**	Abundance of the four *n*-alkanes *n*-C_{27}, *n*-C_{29}, *n*-C_{31} and *n*-C_{33} over the last ~130 kyr.
**Supplementary Fig. S8**	Annual average δD and rainfall amount for the last Interglacial (130 to 115 ka BP) and the middle to late Holocene (7 ka BP to present) for the G-B-M catchment.
**Supplementary Fig. S9**	Transient climate simulation results from Earth system model COSMOS-wiso for the Last Interglacial and the Holocene
**Supplementary Background 1**	Background on the current simulated climate and stable water isotope models.
**Supplementary Discussion 1**	Leaf wax δD as a quantitative proxy for ISM rainfall changes
**Supplementary Discussion 2**	Rainfall patterns during marine isotope stage (MIS) 5c.

Supplementary Table S1. List of model parameters (basis function and smoothing parameter) applied in the Generalized Additive Model (GAM) for different proxies from sediment core SO 188-17286-1 (this study) and sediment core GeoB16602 (*1*). Model check was performed in order to check basis dimension (k) results. δD_{ivc} and $\delta^{13}C$ of the most dominant *n*-alkanes *n*-C_{29} and *n*-C_{31} as well as the concentration-weighted average δD_{ivc} and $\delta^{13}C$ of all four homologues (*n*-C_{27}, *n*-C_{29}, *n*-C_{31}, *n*-C_{33}) were considered for the GAM model.

		**105-130 ka BP**			**20 ka BP-present**		
**Core**	**Proxy**	**k**	**sp**	***p*-value**	**k**	**sp**	***p*-value**
SO 188-17286-1	δD_{ivc} (*n*-C_{29}, *n*-C_{31})	20	0.01	0.68	25	0.01	0.42
SO 188-17286-1	*Weighted average* δD_{ivc}	20	0.01	0.82	20	0.01	0.40
SO 188-17286-1	$\delta^{13}C$ (*n*-C_{29}, *n*-C_{31})	20	0.01	0.69	20	0.01	0.025
SO 188-17286-1	*Weighted average* $\delta^{13}C$	20	0.01	0.98	20	0.01	0.98
SO 188-17286-1	SST	20	0.01	0.32	30	0.001	0.2
SO 188-17286-1	$\delta^{18}O_{sw\text{-}ivc}$	5	0.01	0.87	15	0.01	0.71
GeoB16602	δD	6	0.01	0.14	6	0.01	0.38

Supplementary Table S2. Multivariate analysis of variance (MANOVA) and analysis of variance (ANOVA) results comparing average δD_{ivc} values for the Holocene and the Last Interglacial. Univariate ANOVA performed on the output from MANOVA indicates that the distinct differences between the two interglacial periods are driven by both *n*-C_{29} and *n*-C_{31} (P=0.004 for *n*-C_{29} and P=0.015 for *n*-C_{31}; Supplementary Table S3).

MANOVA summary

	Df	Pillai	Approx. F	Num DF	den DF	P
Period	1	0.27611	5.7214	2	30	0.008
Residuals	31					

ANOVA summary

Response δD_{ivc} of *n*-C_{29}:

	Df	Sum Sq	Mean Sq	F value	P
Period	1	623.97	623.97	9.3994	0.00447
Residuals	31	2057.92	66.38		

Response δD_{ivc} of n-C_{31}:

	Df	Sum Sq	Mean Sq	F value	P
Period	1	296.84	296.844	6.6432	0.01493 *
Residuals	31	1385.21	44.684		

Supplementary Table S3. ANOVA test comparing the $\delta^{18}O_{sw\text{-}ivc}$ values for the Holocene and the Last Interglacial. Our result shows that the $\delta^{18}O_{sw\text{-}ivc}$ values for the two interglacial periods are not significantly different.

ANOVA summary

	Df	Sum Sq	Mean Sq	F value	P
Period	1	1.046	1.0458	2.746	0.113
Residuals	20	7.617	0.3809		

Fig. S1. Sedimentation rate (cm kyr^{-1}) for sediment core SO 188-17286-1 over the last ~130 kyr.

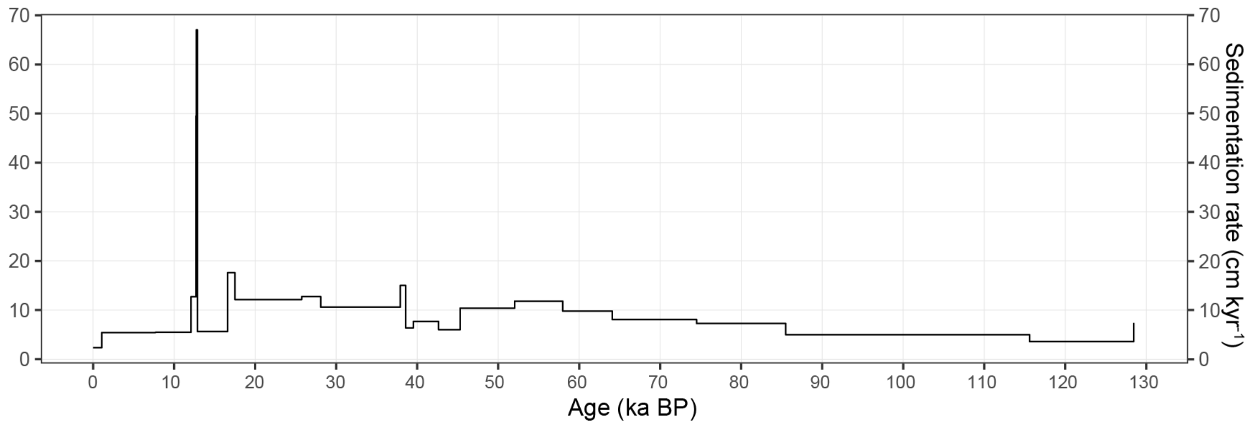

Fig. S2. Uncorrected δD (A) and δ^{13}C (B) values for the four dominant long chain *n*-alkanes *n*-C_{27}, *n*-C_{29}, *n*-C_{31}, and *n*-C_{33} from sediment core SO 188-17286-1.

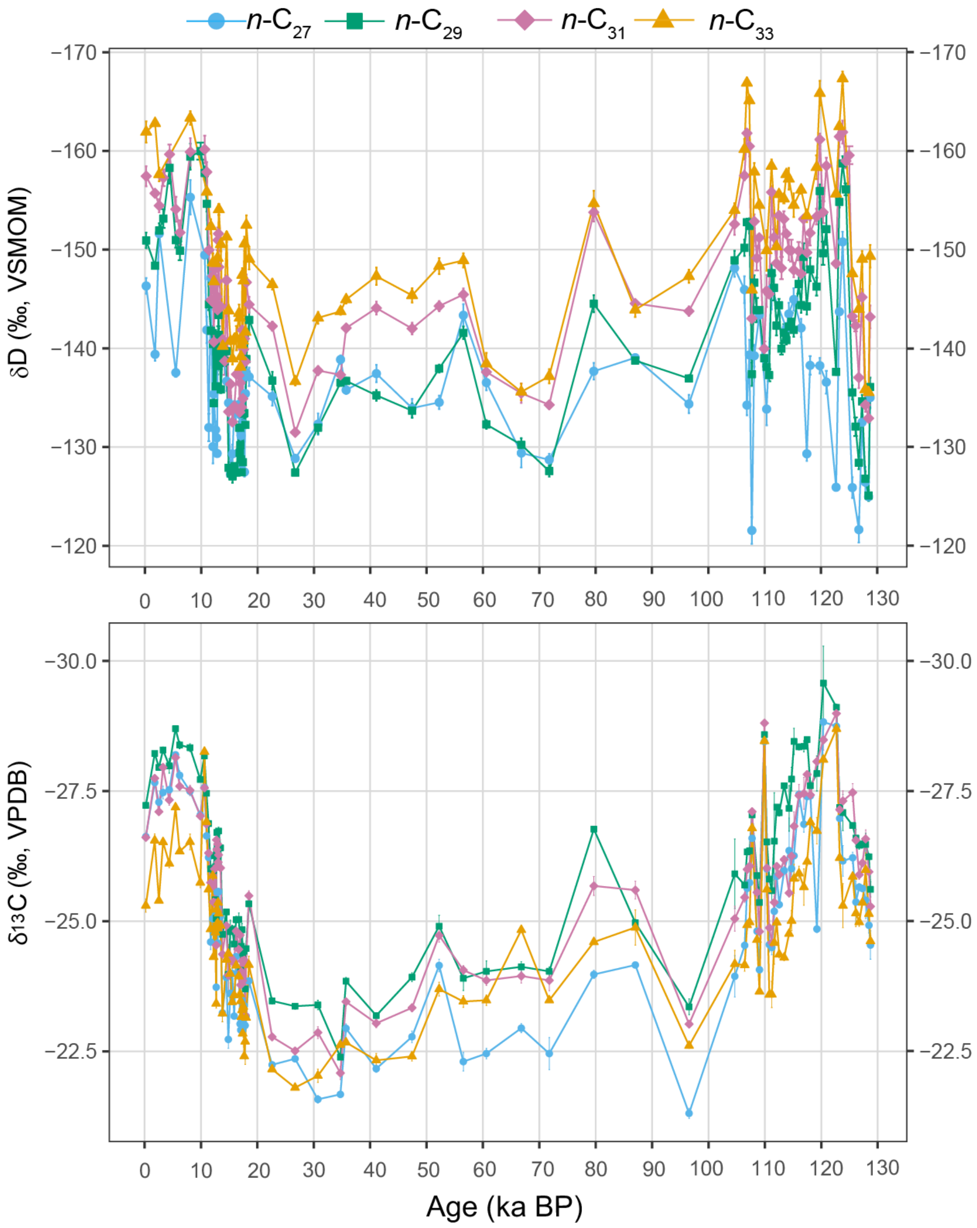

Fig. S3. Ice-volume-corrected δD (δD_{ivc}) values for the four dominant long chain *n*-alkanes *n*-C_{27}, *n*-C_{29}, *n*-C_{31}, and *n*-C_{33} from sediment core SO 188-17286-1.

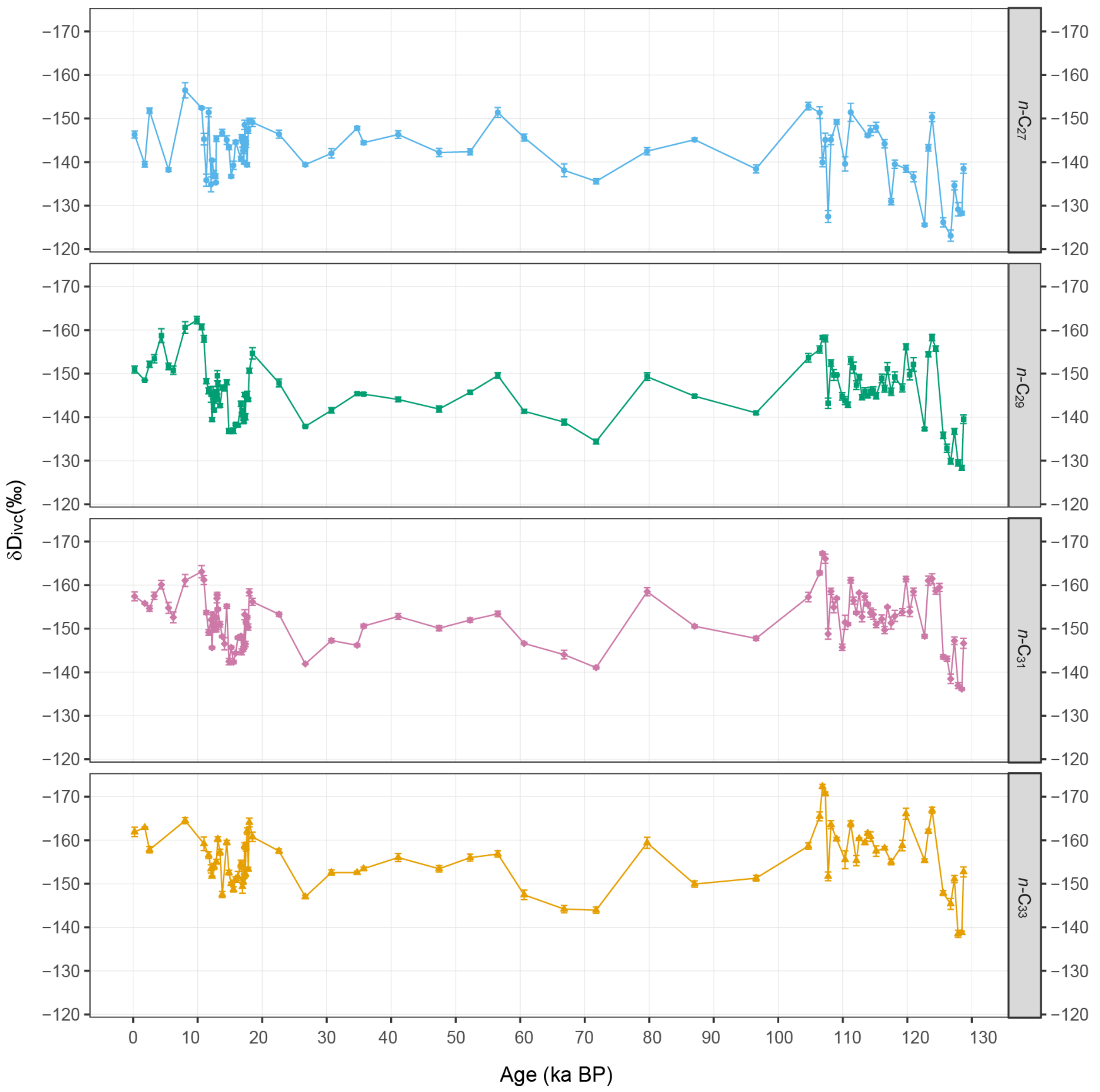

Fig. S4. Correlation between paired δD_{ivc} and $\delta^{13}C$ values for the four *n*-alkanes *n*-C_{27}, *n*-C_{29}, *n*-C_{31}, and *n*-C_{33}. The lack of correlation between δD_{ivc} and $\delta^{13}C$ for each *n*-alkane homologue suggests a limited influence of vegetation type changes (C_4 vs. C_3 plants) on δD_{ivc}.

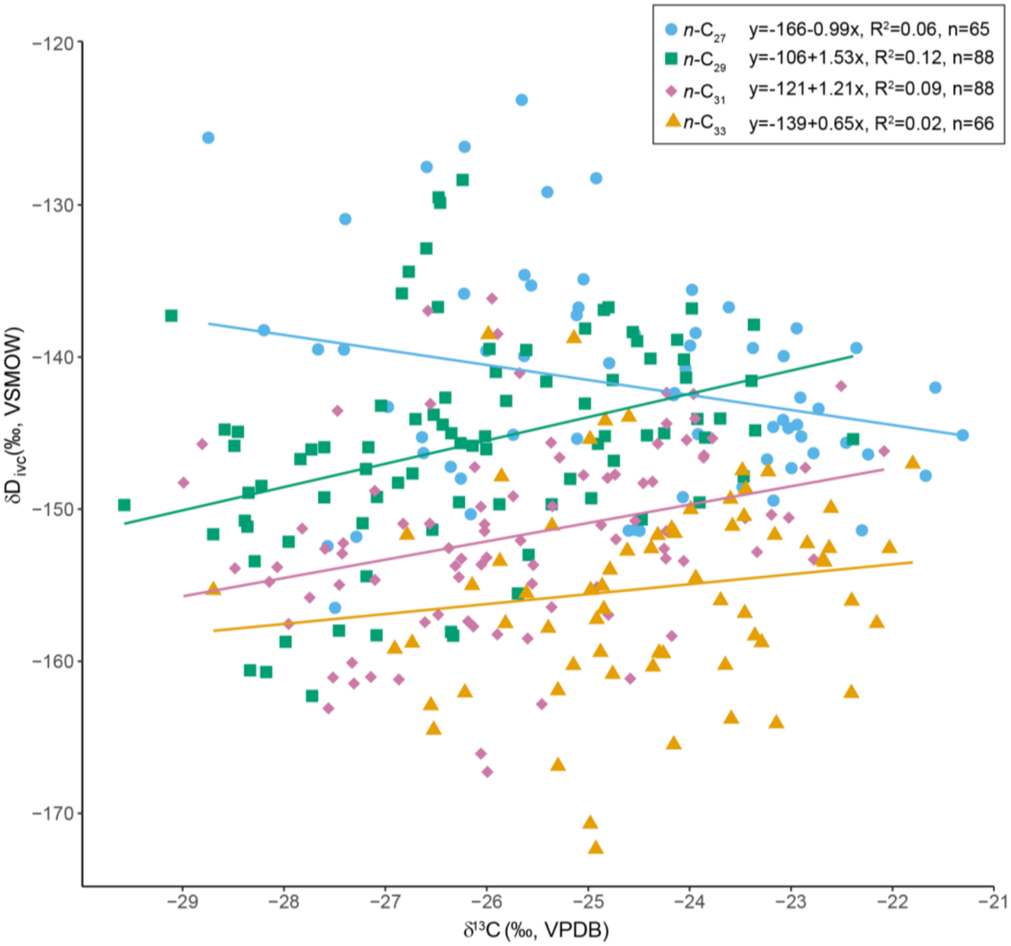

Fig. S5. Correlation between precipitation δD and air temperature for all weather stations located in the G-B-M catchment (meteorological data available at the Global Network of Isotopes in Precipitation; International Atomic Energy Agency (IAEA); http://www.iaea.org/water. Last data access in February 2022). The poor correlation between precipitation δD and air temperature suggests that the temperature effect on precipitation δD is negligible. We also ran a linear regression model to examine whether precipitation amount, air temperature, and the interaction of both affect the δD of precipitation. Our result suggests that precipitation amount and the interaction between precipitation amount and air temperature play a more important role controlling the δD of precipitation than air temperature (P=0.003, 0.0002, and 0.09 for precipitation, precipitation-temperature interaction, and temperature, respectively).

The weather station locations can be found in the main text Fig. 1. These weather stations are: Allahabad, Barisal, Chuadanga, Dhaka (Savar), Dinajpur, New Delhi, Satkhira, Shillong, and Sylhet. The metrological data are shown in Fig. S6.

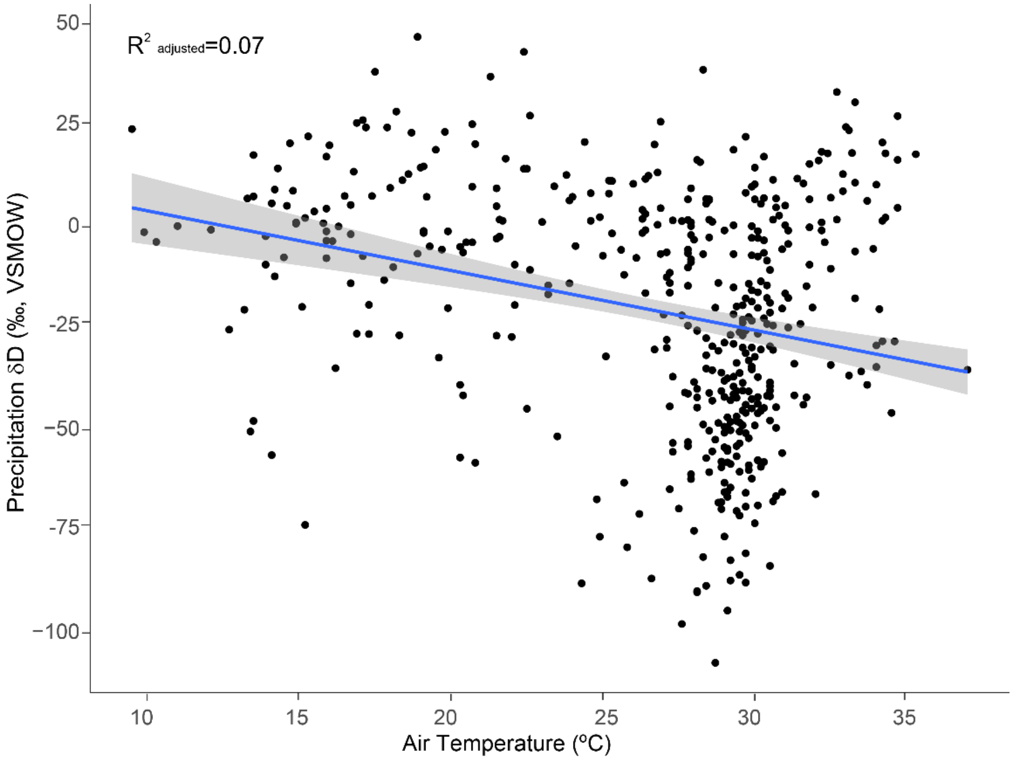

Fig. S6. Monthly mean rainfall (grey bars), air temperature (blue curves) and precipitation δD values (orange curves) for Allahabad, Barisal, Chuadanga, Dhaka (Savar), Dinajpur, New Delhi, Satkhira, Shillong, and Sylhet in the G-B-M catchment (meteorological data available at the Global Network of Isotopes in Precipitation; International Atomic Energy Agency (IAEA); http://www.iaea.org/water. Last data access in February 2022). To ensure data consistency, we only chose data from IAEA weather stations.

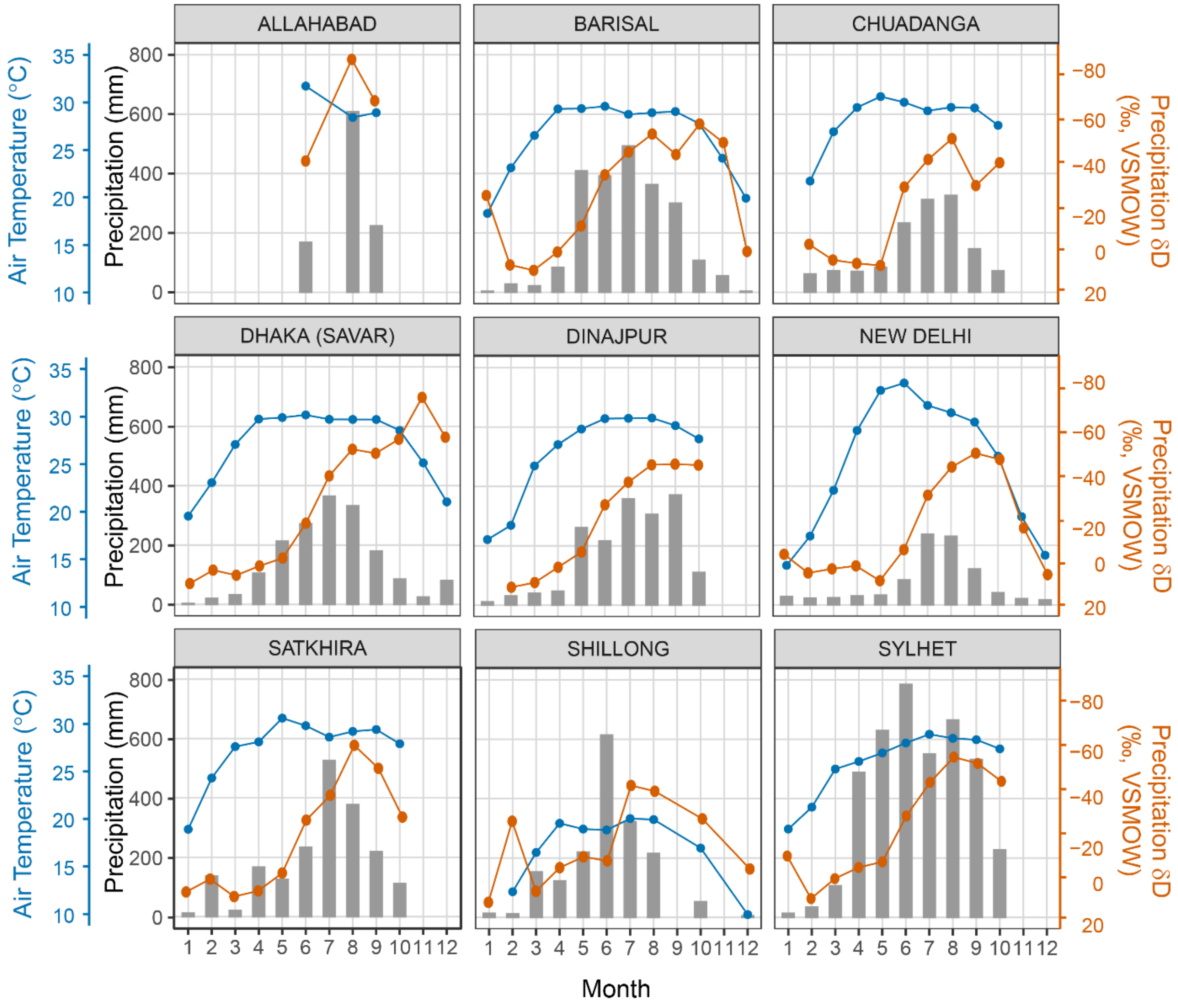

Fig. S7. Abundance of the four *n*-alkanes *n*-C_{27}, *n*-C_{29}, *n*-C_{31} and *n*-C_{33} over the last ~130 kyr. Among the four homologues, *n*-C_{31} and *n*-C_{29} are the most abundant.

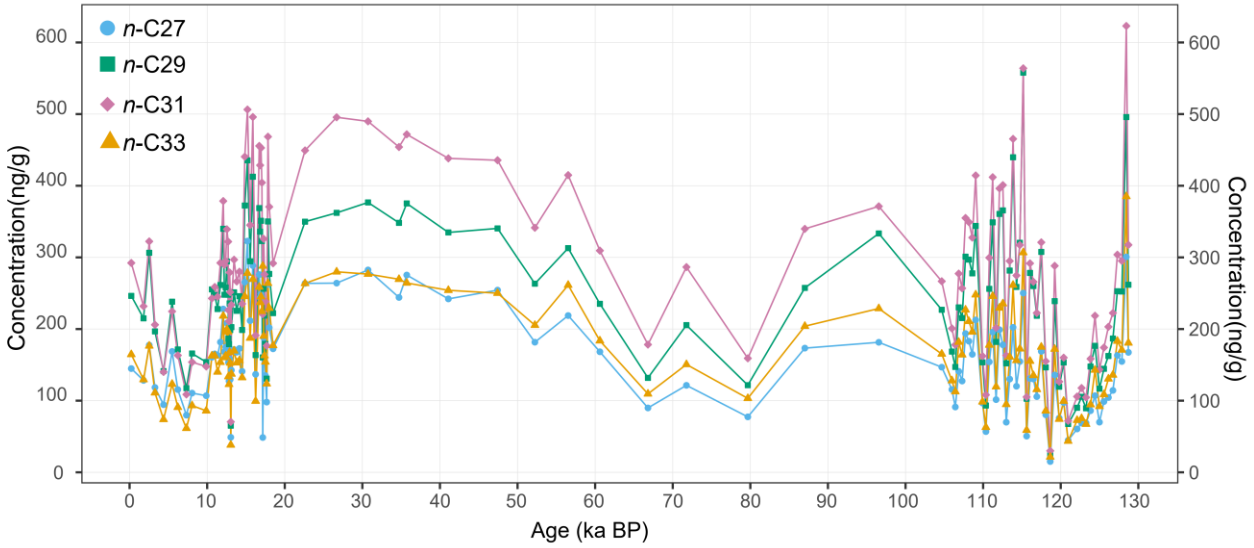

Fig. S8. Two transient simulations of the fully enhanced climate isotope model COSMOS-wiso (*2*). Annual mean δD and total precipitation for a) the mid- to late- Holocene (7 ka BP to present); and b) the Last Interglacial and subsequent northern hemisphere cooling (130 to 115 ka BP) for the G-B-M catchment (21–32°N, 73–99°E).

COSMOS model is fully coupled general circulation model consists of the atmosphere model ECHAM5, the ocean-sea ice model MPI-OM, and the vegetation model JSBACH. COSMOS-wiso is based upon COSMOS yet additionally enables the extension for water stable isotopes.

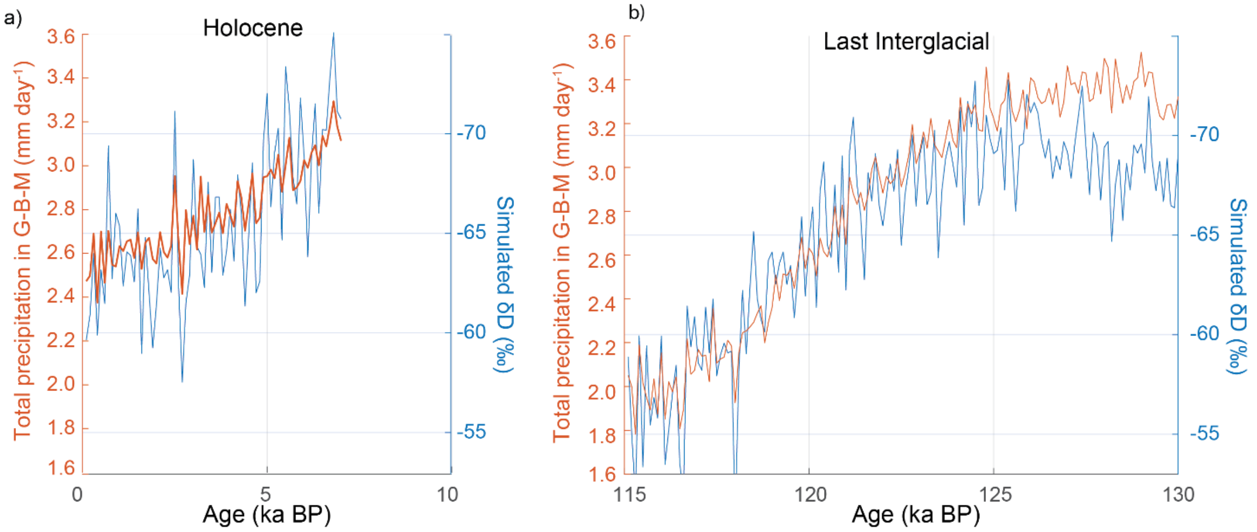

Fig. S9. COSMOS-wiso results from transient experiments (130 to 115 ka BP and 7 ka BP to present): a) relation between total precipitation in the G-B-M catchment (21–32°N, 73–99°E) and sea surface temperature derived at an Indian Ocean grid box (17–21°N, 87–90°E), covering our sediment core location (19°44.58′N, 89°52.76′E); (b) relation between total precipitation in the G-B-M catchment and the convective precipitation above the Indian Ocean grid box that covers our sediment core location. The detailed model experiment set up can be found in (*3*) for the Last Interglacial and in (*4*) for the Holocene.

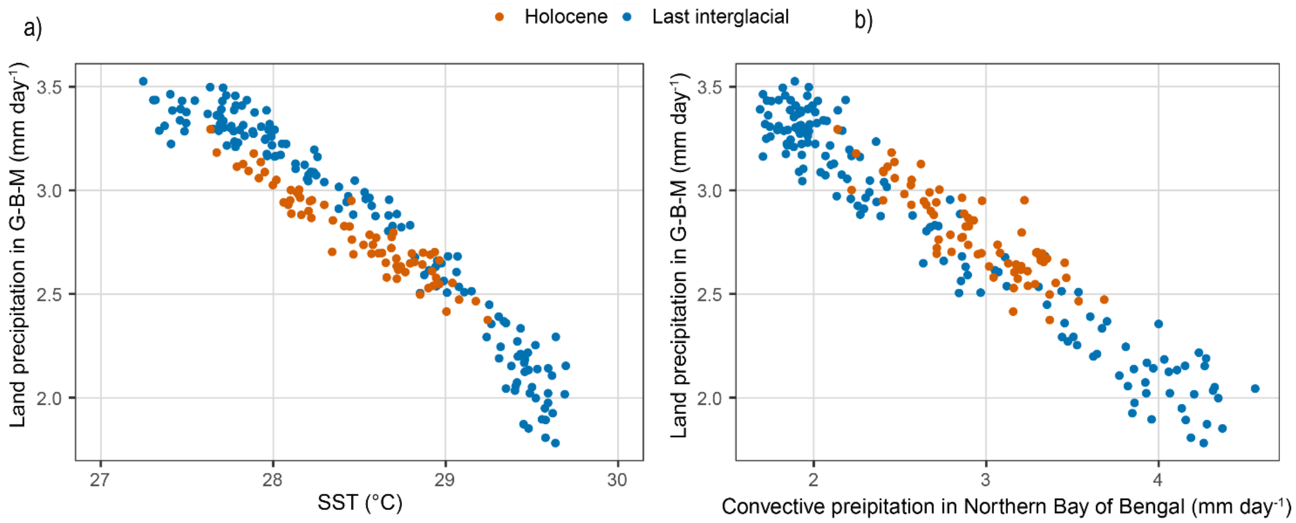

Supplementary Background 1: Background on the current simulated climate and stable water isotope models.

To compare δD_{ivc} values and infer rainfall amount across different warm climate states, we also examined whether the relationship between δD_{ivc} and rainfall amount was similar during the Holocene and the Last Interglacial. The best way to validate this is to use water stable isotope-equipped climate models to investigate the relationships between water isotopes (e.g. $\delta^{18}O$ and δD) and rainfall amount. Several studies have used isotope-enabled climate models to simulate different stages during the Last Interglacial (*5-7*) or Holocene (*8*), consistently showing a strong inverse correlation between water isotope $\delta^{18}O/\delta D$ and rainfall amount in the ISM region at different time slices. For example, one study used the Earth system model ECHAM5/MPIOM to examine the relationships between ISM rainfall and water isotopes at 130, 125 and 120 ka BP at 3.75° × 3.75° grid resolution (*8*) whereas another study used the isotope-enabled model ECHAM4-iso at 2.8° × 2.8° grid resolution (*8*). A newer study examined the relationships between ISM rainfall and water isotopes at different stages during the Holocene (e.g. 0, 2, 4, 6, and 8 ka BP) (*8*).

In order to directly compare the last two interglacials, we analyzed two transient climate simulations of the Last Interglacial (130 to 115 ka BP) (*3*) and the Holocene (7 ka BP to pre-industrial) (*4*) from the coupled climate-isotope model COSMOS-wiso. This fully coupled general circulation model consists of the atmosphere model ECHAM5, the ocean-sea ice model MPI-OM, and the vegetation model JSBACH, with 3.75° × 3.75° grid resolution in the atmosphere and vegetation component, and approximately 1.5° × 3.5° grid resolution in the ocean. Both transient simulations are changing their forcing according to the orbital constellation (*9*) and reconstructed greenhouse gas (GHG) concentrations (*10*) of the respective periods. Both forcing factors however change at a ten-time accelerated rate following a method described by Lorenz and Lohmann (2004) (*11*). These experiments are suitable for a direct comparison between the two periods as the simulations only differ in their transient forcing and particularly allow to test whether the relationship between ISM precipitation and water isotopes remains stable over time. The early Holocene was however not included in the Holocene experiment as remnant ice, deglacial melt water and cold ocean temperatures from the preceding ice age may have additionally influenced the climate. For the same reason the Last Interglacial experiment may be less realistic in its earlier part.

Supplementary Discussion 1: leaf wax δD as a quantitative proxy for ISM rainfall changes

From the COSMOS-wiso model, we find that δD and rainfall amount are strongly inversely correlated during the two interglacials and their relationship remain stable over largest parts of the two interglacials except for the period 130–126 ka BP. During this early part of the Last Interglacial the two variables appear to be decoupled with simulated precipitation gradually decreasing while δD does not display any substantial change (Supplementary Fig. S8a). Interestingly, during the same period, simulated rainfall decreases even though insolation increases, which again points to that insolation may not be the most important factor controlling the rainfall changes in the warm climate state and many different climate forcing factors could also interplay with each other. This decoupling of δD and rainfall amount in the early part of Last Interglacial could either hint at underestimated rainfall amount and/or point towards large scale of hydrologic

changes over the Indian Ocean. The early period of an interglacial might still be affected by the glacial-interglacial transition, which, by design, is not represented in the model setup (*3, 4*). In addition, this decoupling of δD and rainfall amount was not observed in other water isotope models that modelled time slices at 130 and 126 ka BP (*5, 6*), therefore, this weak correlation could be an artefact of model set up.

Nonetheless, it is noteworthy that during the transition into the Last Interglacial, the change rate of our proxy δD is much greater than that of simulated δD signal (when the simulated δD values deviate from modelled precipitation). However, simulated peak δD and precipitation converge at the peak of Last Interglacial (~125ka) (Supplementary Fig. S8b). Once the transient model entered stable interglacial conditions, δD and precipitation amount are strongly correlated, demonstrating similar δD ranges corresponding to similar precipitation estimates with small variations (Supplementary Fig. S8a and b) for the Holocene and Last Interglacial. Furthermore, the observed weak relation in the COSMOS transient simulation from 130 to 126 ka BP doesn't affect our interpretation of δD peaks at 124.5 ka BP because the relationship of δD and rainfall amount is very tight between 126 to 115 ka BP. In addition, the leaf wax δD peak at 124.5 ka BP is comparable to those at 7 ka BP in our data. Taken at face value, this implies that the precipitation at the peak of Last Interglacial should be similar to those at 7 ka BP, which was still lower than the leaf wax δD peak at 11-9 ka BP. Moreover, our proxy data capture the sharp transition at the onset of the Last Interglacial with the rapid change of leaf wax δD more realistically than the model. We take as further evidence that the proxy signal is quite robust in terms of quantifying rainfall amount with respect to the Last Interglacial.

Supplementary Discussion 2: Rainfall patterns during marine isotope stage (MIS) 5c

For MIS 5c, we do not have a high-resolution rainfall proxy records as the high-resolution record only reaches from ~130 to 105 ka BP, i.e. until the onset of MIS 5c. After that, the rainfall proxy record has a much lower resolution, which is largely due to a change in the sampling interval at the onset of MIS 5c as our primary aim for this study was to compare the rainfall patterns during the Last Interglacial and the Holocene.

Although it seems that ISM intensity inferred from the concentration-weighted average δD_{ivc} is out of phase with summer insolation at ~103 ka BP (onset of MIS 5c) and that peak ISM intensity leads peak insolation (Fig. 2c in the main text), we argue that this is in fact not the case for several reasons. First, the δD_{ivc}-inferred rainfall peak occurred around 106–107 ka BP, whereas the summer insolation maximum occurred at 103 ka BP. This time difference is within the 1σ age age uncertainty range of the sediment core chronology (4.4 kyr) (*12*). Second, the δD_{ivc}-inferred rainfall record (Fig. 2c in the main text) mimics changes in the $\delta^{18}O_{sw\text{-}ivc}$ (Fig. 2d in the main text), which is synchronous to summer insolation. Third, peak ISM intensity at ~106-107 ka BP (Fig. 2c in the main text) coincides with peak intensity of the East Asian Summer Monsoon (i.e. Hulu and Dongge Cave $\delta^{18}O$) for the same period (Fig. 2e in the main text) (*13*). The sharp decreases in rainfall recorded by cave $\delta^{18}O$ at 105 ka BP also coincide with rapid drops in rainfall (i.e. increases in δD values) at the same time in our record. On the basis of the $\delta^{18}O_{sw\text{-}ivc}$ curve, we suppose that the

δD_{ivc}-inferred MIS 5c rainfall peak could have actually lasted from 107 to 100 ka BP, which would be longer than the interval covered by the high-resolution data.

Reference

1. E. Huang *et al.*, Precession and glacial-cycle controls of monsoon precipitation isotope changes over East Asia during the Pleistocene. *Earth and Planetary Science Letters* **494**, 1-11 (2018).
2. J. H. Jungclaus *et al.*, Ocean Circulation and Tropical Variability in the Coupled Model ECHAM5/MPI-OM. *Journal of Climate* **19**, 3952-3972 (2006).
3. R. Stein, K. Fahl, P. Gierz, F. Niessen, G. Lohmann, Arctic Ocean sea ice cover during the penultimate glacial and the last interglacial. *Nat. Commun.* **8**, 373 (2017).
4. C. Danek *et al.*, Eurasian Holocene climate trends in transient coupled climate simulation and stable isotope records. *Journal of Quanterary Science*, Accepted (2021).
5. P. Gierz, M. Werner, G. Lohmann, Simulating climate and stable water isotopes during the Last Interglacial using a coupled climate-isotope model. *Journal of Advances in Modeling Earth Systems* **9**, 2027-2045 (2017).
6. J. Sjolte, G. Hoffmann, Modelling stable water isotopes in monsoon precipitation during the previous interglacial. *Quaternary Sci Rev* **85**, 119-135 (2014).
7. M. Herold, G. Lohmann, Eemian tropical and subtropical African moisture transport: an isotope modelling study. *Climate Dynamics* **33**, 1075-1088 (2009).
8. T. Tharammal *et al.*, Orbitally driven evolution of Asian monsoon and stable water isotope ratios during the Holocene: Isotope-enabled climate model simulations and proxy data comparisons. *Quaternary Sci Rev* **252**, 106743 (2021).
9. A. L. Berger, Long-Term Variations of Daily Insolation and Quaternary Climatic Changes. *Journal of the Atmospheric Sciences* **35**, 2362-2367 (1978).
10. P. Köhler, C. Nehrbass-Ahles, J. Schmitt, T. F. Stocker, H. Fischer, A 156 kyr smoothed history of the atmospheric greenhouse gases CO2, CH4, and N2O and their radiative forcing. *Earth Syst. Sci. Data* **9**, 363-387 (2017).
11. S. J. Lorenz, G. Lohmann, Acceleration technique for Milankovitch type forcing in a coupled atmosphere-ocean circulation model: method and application for the Holocene. *Climate Dynamics* **23**, 727-743 (2004).
12. S. Lauterbach *et al.*, An ~130 kyr Record of Surface Water Temperature and δ18O From the Northern Bay of Bengal: Investigating the Linkage Between Heinrich Events and Weak Monsoon Intervals in Asia. *Paleoceanography and Paleoclimatology* **35**, e2019PA003646 (2020).
13. H. Cheng *et al.*, The Asian monsoon over the past 640,000 years and ice age terminations. *Nature* **534**, 640-646 (2016).
